# Long-Term Impacts of Foetal Malnutrition Followed by Early Postnatal Obesity on Fat Distribution Pattern and Metabolic Adaptability in Adult Sheep

**DOI:** 10.1371/journal.pone.0156700

**Published:** 2016-06-03

**Authors:** Prabhat Khanal, Lærke Johnsen, Anne Marie Dixen Axel, Pernille Willert Hansen, Anna Hauntoft Kongsted, Nette Brinch Lyckegaard, Mette Olaf Nielsen

**Affiliations:** Department of Veterinary Clinical and Animal Sciences, Faculty of Health and Medical Sciences, University of Copenhagen, Frederiksberg, Denmark; The University of Manchester, UNITED KINGDOM

## Abstract

We aimed to investigate whether over- versus undernutrition in late foetal life combined with obesity development in early postnatal life have differential implications for fat distribution and metabolic adaptability in adulthood. Twin-pregnant ewes were fed NORM (100% of daily energy and protein requirements), LOW (50% of NORM) or HIGH (150%/110% of energy/protein requirements) diets during the last trimester. Postnatally, twin-lambs received obesogenic (HCHF) or moderate (CONV) diets until 6 months of age, and a moderate (obesity correcting) diet thereafter. At 2½ years of age (adulthood), plasma metabolite profiles during fasting, glucose, insulin and propionate (in fed and fasted states) tolerance tests were examined. Organ weights were determined at autopsy. Early obesity development was associated with lack of expansion of perirenal, but not other adipose tissues from adolescence to adulthood, resulting in 10% unit increased proportion of mesenteric of intra-abdominal fat. Prenatal undernutrition had a similar but much less pronounced effect. Across tolerance tests, LOW-HCHF sheep had highest plasma levels of cholesterol, urea-nitrogen, creatinine, and lactate. Sex specific differences were observed, particularly with respect to fat deposition, but direction of responses to early nutrition impacts were similar. However, prenatal undernutrition induced greater metabolic alterations in adult females than males. Foetal undernutrition, but not overnutrition, predisposed for adult hypercholesterolaemia, hyperureaemia, hypercreatinaemia and hyperlactataemia, which became manifested only in combination with early obesity development. Perirenal expandability may play a special role in this context. Differential nutrition recommendations may be advisable for individuals with low versus high birth weights.

## Introduction

In the early 1990’ies it was demonstrated that prenatal undernutrition and low birth is associated with permanent changes in metabolic and endocrine function, which predisposes for metabolic disorders later in life [1]. Later on, high-birth-weight was shown to be associated with similar disease risk implications later in life, and a ‘U’–shaped curve was established relating birth weight to the risk of adult obesity and metabolic disorders [2, 3]. This suggests that widely different nutritional insults (over- versus undernutrition) during gestation may predispose individuals born at the extremes of the birth weight spectrum for similar adverse disease outcomes later in life. In a previous study using sheep as an experimental model, we have confirmed that both prenatal over- and undernutrition during late gestation predisposes for development of abdominal obesity also in adolescent lambs, but we found indications to suggest that the underlying physiological adaptations may be different, since prenatal overnutrition predisposed for adolescent hyperglycaemia and hyperlactataemia, whereas prenatal undernutrition predisposed for hypercholesterolaemia, which became evident upon subsequent exposure to an early postnatal obesogenic diet [4, 5].

Other studies have also shown that the diet received in early postnatal life may have implications for the development as well as severity of disorders associated with a prenatal malnutrition exposure. For example, postnatal consumption of a high-fat diet induced mitochondrial dysfunction in skeletal muscle in intrauterine growth restricted pig offspring [6]. Increased food intake and sedentary behaviour has been reported in prenatally undernourished rat offspring, and this was further amplified by exposure to a hypercaloric diet after weaning [7, 8]. In Sprague Dawley rats, a high-fat cafeteria diet for 5-week before mating and throughout gestation and lactation led to adiposity, glucose intolerance, hyperlipidaemia and reduced hypothalamic neuropeptide Y and increased proopiomelanocortin mRNA expression in offspring, and this was amplified by postnatal overnutrition [9].

Few studies have been conducted to show whether adverse outcomes of different pre- and early postnatal malnutrition exposures can be rescued or alleviated by dietary intervention later in life. In rats, pups weaned to high-multivitamin or high-folate diets were prevented from developing the obesogenic phenotype (higher food intake, body weight and glucose response to a glucose load) induced by maternal exposure to a high-multivitamin diet during pregnancy [10]. In our previous sheep studies, we have observed that when obesity was induced in early postnatal life in lambs with different prenatal nutrition histories, dietary correction from puberty until adulthood could reverse the majority of the adverse impacts of the early postnatal obesogenic diet on liver and muscle function [11, 12]. However, late gestational undernutrition irrespectively of the postnatal diet permanently depressed whole-body insulin sensitivity in adolescent male and female lambs [13] and induced alterations in a number of endocrine systems under hypothalamic control in adult female sheep [14, 15], and reduced thickness of the subcutaneous fat (SUBF) layer in both male and female animals [16]. It is not known, whether dietary correction to correct for early postnatal obesity development can alleviate the long-term outcomes of prenatal undernutrition in males or of overnutrition in either males or females. Such information must be generated to evaluate the potential need for differential dietary recommendations for individuals born at the extremes of the birth weight spectrum.

We hypothesized that 1) under- and overnutrition in late foetal life have differential long-term implications for fat distribution patterns and metabolic adaptability in adulthood and 2) long-term implications of obesity development in early postnatal life are more extensive in individuals with a history of prenatal undernutrition as compared to overnutrition. To test these hypotheses, we conducted a study in the Copenhagen sheep model [4] with 2½-year old adult sheep, which had been exposed to under- (LOW) or overnutrition (HIGH) in late foetal life, fed a moderate (CONV) or an obesogenic (HCHF) diet from birth until 6-months of age (around puberty), and thereafter fed a moderate (and for obese sheep: body fat correcting) diet from 6 months until 2½ years of age (adulthood). Metabolic adaptabilities in adulthood were assessed in different trials reflecting situations of nutrient scarcity (3-day fasting period) and acute nutrient surplus (*in-vivo* glucose, insulin and propionate tolerance tests). Organ and tissue weights were thereafter determined at autopsy.

## Materials and Methods

The present experiment was conducted at the experimental facilities on the farm Rosenlund, Lynge, Denmark under the auspices of the Faculty of Health and Medical Sciences, University of Copenhagen, Frederiksberg, Denmark. All experimental procedures were approved by the National Committee on Animal Experimentation, Denmark.

### Experimental animals and design

The Copenhagen sheep model was used in this experiment, and details about the experimental animals and design until 6-months of age (around puberty) have been described in a previous paper [4]. In short, the experiment was a 3×2 factorial design, where 36 twin-pregnant Texel ewes were assigned to one of three feeding treatments during the last six weeks of gestation (term = 147 days): NORM (N = 9; fulfilling 100% of recommended daily allowances for digestible energy and protein), HIGH (N = 13; 150% of digestible energy and 110% of protein provided to NORM), LOW (N = 14; 50% of digestible energy and protein provided to NORM). After lambing, the two siblings were assigned to separate diets, either a conventional (CONV; N = 35; 16 males, 19 females) or an obesogenic high-carbohydrate-high-fat (HCHF; N = 35; 18 males, 17 females) diet from 3-days until 6-months of age. The CONV diet consisted of hay supplemented by a milk replacer during the first 8-weeks of life, and daily allowances were adjusted weekly to ensure constant and moderate daily weight gain (appr. 225 g/day). The HCHF lambs were fed a mixture of 50% milk replacer and 50% dairy cream (max. 2.5 L per day) and rolled maize (max. 1 kg/day). At six months of age, sub-groups of animals were slaughtered as previously reported [4]. Remaining animals were kept until adulthood for this study. Additionally, eight (4 males; 4 females) age-matched lambs (6-months of age) were obtained from the Danish commercial sheep farm, which delivered the pregnant dams that gave birth to the other experimental lambs, and these lambs were included in this experiment as undisturbed, external controls (EC) from 6 months to 2½ years of age. This resulted in seven different groups: HIGH-HCHF, HIGH-CONV, LOW-HCHF, LOW-CONV, NORM-HCHF, NORM-CONV, and EC. All animals were raised in two (sex divided; males remained uncastrated) flocks from 6-months until 2½-years (adulthood) of age, and received the same moderate diet consisting of high-quality grass hay *ad libitum* supplemented during the first months with rolled barley. Water and a commercial vitamin-mineral mix was available *ad libitum* at all times. At 2½-years of age, the sheep were transferred to individual saw dust bedded pens (1.5 × 1.5 m) to allow conduct of tolerance tests (see below). Body weights were recorded monthly from 6-months until 2½-years of age and body proportions (crown-rump length, girth circumference, height over withers, head width) at 6-months, 1½- and 2½-years of age.

### Catheterization and blood sampling

Permanent indwelling catheter tubes were placed in both jugular veins at least one day prior to the first tolerance test to facilitate repeated collection of blood samples as described by Khanal et al. [5] except that a silicone tubing (Silicone Rubber Tube, 1.2 mm ID × 0.4 mm wall; Silex Ltd., Lindford, Bordon Hampshire UK) was used as the indwelling permanent catheter material. Blood samples were collected at different time points depending upon the tolerance test (see below) and all blood samples were collected in EDTA tubes and processed as previously described [5].

### Fasting and intra-venous tolerance tests

A fasting tolerance test was conducted as described by Khanal et al. [4], except that the fasting period had a duration of 68 hours and blood samples were collected at 13:00 (0 sample), 24, 48 and 68 hour after the onset of fasting (FTT). All animals were provided water *ad libitum* all the time during fasting. *In vivo* tolerance tests with acute intravenous bolus injections of glucose (conducted in the morning prior to feeding the morning meal in overnight fasted sheep; GTT), insulin (conducted at mid-day appr. 5 hours after feeding; ITT) and propionate (one conducted after 68 hours of fasting; PPT-fasted and one conducted in the fed state right after feeding the morning meal; PPT-fed) were conducted as described previously [5].

### Laboratory analyses

Glucose, non-esterified fatty acids (NEFA), triglycerides (TG), blood urea nitrogen (BUN), creatinine, lactate, β-hydroxy-butyrate (BOHB), γ-glutamyl transferase (GGT) and cholesterol levels were determined in plasma samples as previously described [4, 5]. The intra- and interassay coefficients of variation were below 5 and 10%, respectively, for all assays.

### Slaughtering

At 2½ years of age, the 44 sheep in total [NORM-CONV: 6 (2 males, 4 females); NORM-HCHF: 4 (2 males, 2 females); HIGH-CONV: 5 (2 males, 3 females); HIGH-HCHF: 6 (3 males, 3 females); LOW-CONV: 9 (4 males, 5 females); LOW-HCHF: 7 (4 males, 3 females); EC: 7 (3 males, 4 females)] were anaesthetized and sacrificed by decapitation, and different organs excised and weighed as previously described [4].

### Statistical analyses

Statistical evaluation of all data was performed in SAS (v.9.2; SAS Institute, USA). Homogeneity of variance was evaluated by visual inspection of residual plots and normality of residuals was tested by means of quantile-quantile plots. Log transformations were applied when it was needed to obtain normal distribution of residuals. Analyses for body weights, body proportions, weights of different organs and tissues and for different parameters during the tolerance tests were performed using the generalized linear mixed model (PROC GLIMMIX) procedure by two different approaches: either including all animals divided in seven individual experimental groups, or excluding the EC animals to be able to compare separate and interactive effects of pre- and postnatal nutrition and sex. For the repeated measurements, different correlation structures between measurements and inhomogeneous variances were tested and the structure yielding the best fit was chosen. The models included *fixed effects* of pre- and early postnatal nutrition levels, sex and time of sampling and their interactions; individual sheep within feeding level were included as *random effects*, and samples within sheep were considered *repeated measurements*. Difference in least square means (LS means) were compared by Tukey’s multiple comparison test, and the presented results are expressed as LS means with standard error of means (LS means±SEM).

## Results

We have previously reported how prenatal malnutrition affected birth weights (HIGH: 4.38±0.15; NORM: 4.35±0.18; LOW: 3.89±0.15 kg) and pre- and postnatal nutrition altered body weight and body condition score (BCS) [4], metabolic responses during glucose, insulin, propionate and fasting tolerance tests [5] in sheep included in this experiment, and tissue/organ weights for another subgroup of sheep slaughtered at 6 months of age [4], i.e. when the differential postnatal feeding came to an end. Comparisons will be made when relevant to these results to illustrate changes over the following two years, where all animals received the same moderate diet.

Unless specifically stated in the following, we did not for any of the parameters in this study detect significant effects of pre- or postnatal nutrition, sex, interactions between pre- and postnatal nutrition or sex, or of time of blood sampling during fasting and tolerance tests. It should be noted that due to an uneven distribution of male or female animals in some of the experimental groups, sex differences may not have been sufficiently detected in this experiment.

### Body weight and proportions, organ weights and fat deposition patterns: early life nutrition impacts

From 6 months of age, HCHF and CONV sheep gradually attained similar body weights (S1 Fig) and BCS (*P*<0.0002 for the postnatal diet and time interaction with EC included; results not shown), except for the LOW sheep. LOW-HCHF and LOW-CONV sheep grew to become the heaviest (together with EC) and the lightest (together with NORM-CONV), respectively (Table 1 and S1 Fig; *P<*0.05 for the prenatal nutrition and age interaction). Body weight (Table 1) was consistently highest for EC.

**Table 1:** Effects of pre- and postnatal nutrition and sex on organ and adipose tissue weights in 2½ year old sheep.

Parameters (actual weight, g or % of total body weight)	Treatments groups based on the combinations of pre- and postnatal nutrition*							Sex		*P* values			
	HIGH-HCHF	HIGH-CONV	LOW-HCHF	LOW-CONV	NORM-HCHF	NORM-CONV	EC	Male	Female	ED	LD	Sex	ED*LD
Body weight, kg	94.7±2.9^bc^	98.1±3^abc^	99±2.7^ab^	91.2±2.4^c^	92.1±3.5^bc^	91.13±2.9^c^	103.3±2.7^a^	99.4±1.2^a^	92.9±1.4^b^	0.092	0.92	0.0004	0.011
Subcutaneous fat, g	540±96	686±97.7	688±88.9	554±84.6	726±117.6	535±96	833±88.9	358±52.6^b^	892±48^a^	0.92	0.14	<0.0001	0.25
Subcutaneous fat %	0.59±0.09	0.71±0.10	0.69±0.09	0.60±0.08	0.78±0.12	0.60±0.10	0.82±0.09	0.36±0.05^b^	0.95±0.05^a^	0.98	0.15	<0.0001	0.19
Mescenteric fat, g	3736±524	3854±523	3698±486	2686±453	3939±642	3019±524	3899±485	2632±287^b^	4227±262^a^	0.71	0.035	0.0005	0.55
Mescenteric fat %	4.0±0.52	4.0±0.57	3.7±0.48	2.9±0.43	4.3±0.64	3.4±0.52	3.8±0.48	2.6±0.30^b^	4.6±0.26^a^	0.62	0.034	<0.0001	0.71
Perirenal fat, g	1551±263^b^	2542±265^a^	1715±245^b^	2152±230^ab^	1888±325^ab^	1831±265^b^	2586±243.8^a^	1252±144^b^	2724±132^a^	0.59	0.29	<0.0001	0.033
Perirenal fat %	1.67±0.27	2.65±0.29	1.71±0.25	2.37±0.22	2.07±0.33	2.04±0.27	2.52±0.25	1.20±0.2^b^	2.90±0.10^a^	0.81	0.17	<0.0001	0.055
Sternal fat, g	253±48	261±48	243±44	194±41	212±59	215±48	316±44	142±26^b^	327±24^a^	0.6	0.39	<0.0001	0.79
Sternal fat %	0.27±0.05	0.27±0.05	0.24±0.05	0.21±0.04	0.23±0.06	0.24±0.05	0.31±0.05	0.14±0.03^b^	0.35±0.02^a^	0.68	0.41	<0.0001	0.98
Pericardial fat, g	166±20	227±21	201±19	177±18	218±25	212±25	218±19	178±11^b^	220±11^a^	0.59	0.62	0.02	0.12
Pericardial fat %	0.18±0.02	0.23±0.02	0.20±0.02	0.20±0.02	0.24±0.02	0.24±0.03	0.21±0.02	0.18±0.01^b^	0.24±0.10^a^	0.32	0.59	0.0008	0.26
Thyroids, g	4.7±0.81	6.1±0.90	4.9±0.75	6.1±0.70	5.2±0.99	6.2±0.81	5.9±0.81	5.6±0.50	5.6±0.40	0.94	0.08	0.78	0.92
Thyroids %	0.005±0.001	0.006±0.001	0.005±0.001	0.007±0.001	0.006±0.001	0.007±0.001	0.006±0.001	0.006±0.001	0.006±0.005	0.93	0.12	0.77	0.94
Adrenals, g	3.1±0.5	4.5±0.5	4.1±0.5	4.2±0.4	3.0±0.6	4.0±0.5	4.7±0.5	3.6±0.3^b^	4.4±0.3^a^	0.42	0.062	0.03	0.29
Adrenals %	0.003±0.0005	0.005±0.0006	0.004±0.0005	0.005±0.0004	0.003±0.0007	0.004±0.0005	0.005±0.0005	0.004±0.0003^b^	0.005±0.0003^a^	0.41	0.053	0.027	0.78
Pancreas, g	71.4±11.0	64.9±11.0	68.3±10.2	74±10.0	83.2±13.5	86±11.0	69.5±10.2	79.4±6.2^a^	68.2±5.5^b^	0.31	0.83	0.006	0.85
Pancreas %	0.077±0.012	0.065±0.013	0.069±0.010	0.080±0.010	0.090±0.015	0.094±0.012	0.067±0.011	0.080±0.007	0.07±0.006	0.19	0.9	0.40	0.53
Liver, g	808±35.5	824±35.7	846±33.0	797±33.2	745±43.5	805±35.5	932±35.5	902±20.0^a^	765±18.2^b^	0.48	0.29	<0.0001	0.2
Liver %	0.85±0.03	0.84±0.03	0.86±0.03	0.86±0.03	0.81±0.04	0.88±0.03	0.89±0.03	0.90±0.02^a^	0.80±0.01^b^	0.88	0.2	0.0004	0.29
Kidney, g	160±8.8	155±8.8	169±8.1	178±7.6	156±10.9	164±8.9	177±8.9	181±4.9^a^	155±4.6^b^	0.37	0.36	<0.0001	0.78
Kidney %	0.17±0.01	0.16±0.01	0.17±0.01	0.20±0.01	0.17±0.01	0.18±0.01	0.17±0.01	0.18±0.01^a^	0.17±0.01^b^	0.15	0.3	0.03	0.15
Heart, g	347±19.1	319±19.4	327±17.6	303±16.8	320±27.1	340±19.1	329±17.6	350±10.8^a^	305±9.5^b^	0.23	0.9	0.001	0.46
Heart %	0.37±0.02	0.32±0.02	0.33±0.02	0.33±0.02	0.34±0.03	0.37±0.02	0.32±0.02	0.35±0.01	0.33±0.01	0.36	0.92	0.09	0.22
*Longissimus dorsi*, g	1110±60	1218±59	1193±56	1079±52	1088±73	1037±60	1166±56	1250±33^a^	1029±30^b^	0.27	0.71	<0.0001	0.13
*Longissimus dorsi* %	1.18±0.06	1.24±0.06	1.21±0.05	1.19±0.05	1.18±0.07	1.14±0.06	1.13±0.05	1.30±0.03^a^	1.10±0.03^b^	0.8	0.75	0.003	0.85
*Biceps femoris*, g	666±37	749±36	723±34	672±32	625±45	688±37	743±34	734±20^a^	669±19^b^	0.44	0.17	0.02	0.17
*Biceps femoris* %	0.71±0.04	0.77±0.04	0.73±0.03	0.74±0.03	0.68±0.04	0.75±0.04	0.72±0.03	0.74±0.01	0.72±0.01	0.73	0.12	0.45	0.76

Data are presented as least square means±SEM. Effects of prenatal nutrition, postnatal nutrition or sex were significant (*P*<0.05) if the data within a row and within the respective columns are marked by different superscripts. ED, ewe diet; LD, lamb diet; NORM (N = 10; 4 males, 6 females), normal diet fulfilling requirements for energy and protein; HIGH (N = 11; 5 males, 6 females), 150% of requirements for energy and 110% of requirements for protein; LOW (N = 16; 8 males, 8 females), 50% of requirements for energy and protein; HCHF (n = 13; 8 males, 5 females), high carbohydrate-high fat diet from birth until six months of age and hay-based normal diet thereafter until 2½ years of age; CONV (N = 13; 7 males, 6 females) conventional diet to achieve moderate and constant growth rates of appr. 225 g day-1 from birth until six months of age and hay-based normal diet thereafter until 2½ years of age; EC, external controls (N = 7; 3 males, 4 females). NORM-CONV (N = 6; 2 males, 4 females); NORM-HCHF (N = 4; 2 males, 2 females); HIGH-CONV (N = 5; 2 males, 3 females); HIGH-HCHF (N = 6; 3 males, 3 females); LOW-CONV (N = 9; 4 males, 5 females); LOW-HCHF (N = 7; 4 males, 3 females); EC (N = 7; 3 males, 4 females). The subcutaneous fat represents the fat layer above the *longissimus dorsii* from the right side of the animal. There were interactive effects of prenatal nutrition and sex on body weight, gross and body weight corrected weights of mesenteric and perirenal fat and gross weights of pancreas, kidney and heart as shown in S2 Table. There was an interaction of postnatal nutrition on body weight corrected adrenals as mentioned in the results section previously

Even after being fed the same moderate diet for 2 years, mesenteric fat (MESF) content (% of body weight) was higher in HCHF compared to CONV (*P =* 0.035 with EC, Table 1; *P =* 0.02 without EC). The difference between MESF weights in HCHF and CONV sheep was, however, much smaller in adults (~20% across prenatal groups) as compared to 6 months old lambs, where a 6-fold difference was observed between HCHF and CONV [4].

Perirenal fat (PRF) deposition was not affected by the early postnatal diet in adult NORM sheep, but surprisingly PRF content was reduced by the HCHF diet in HIGH (from 2.65 to 1.67% of body weight) and LOW (from 2.37 to 1.71% of body weight) sheep (interaction of ewe and lamb diet: *P* = 0.033 with EC, Table 1; *P =* 0.064 without EC). This is opposite to what was observed in lambs at 6-months of age, where HCHF had 10-fold more PRF than CONV. In fact, from 6-months to 2½ years of age, there was no expansion of PRF weight at all in HCHF sheep, and the relative content of PRF in the body was thus more than halved in HCHF adults compared to HCHF lambs as body weights were almost doubled (S1 Table). In all the other adipose tissues (including SUBF), tissue weights increased 2–3 fold in HCHF sheep from 6 months to 2½ years of age, and in CONV sheep weights of all adipose tissues (including PRF) were increased 12-16-fold in adults over that observed in lambs [4] (Table 1, S1 Table).

The lack of expansion of PRF weight from adolescence into adulthood in HCHF sheep was reflected in higher deposition ratios of SUBF-to-PRF (*P* = 0.009 with EC; *P =* 0.004 without EC) and MESF-to-PRF (*P<*0.0001 with or without EC) compared to CONV sheep (Table 2) and altered intra-abdominal fat distribution. Thus, MESF constituted 10%-units more of intra-abdominal fat (MESF+PRF) in HCHF (70%) compared to CONV (60%) sheep (Table 2). The intra-abdominal fat distribution was also affected by the prenatal diet, where the proportion of MESF in LOW sheep was slightly reduced (*P* = 0.019 without EC; Table 2).

**Table 2:** Effects of pre- and postnatal nutrition and sex on fat ratios in 2½ year old sheep.

Fat ratios	Treatments groups based on postnatal nutrition			Sex		*P* values			
	HCHF	CONV	EC	M	F	ED	LD	Sex	SD*LD
SUBF:PRF	0.39±0.03^a^	0.27±0.03^b^	0.32±0.05^ab^	0.31±0.03	0.33±0.03	0.87	0.009	0.46	0.83
SUBF:MESF	0.17±0.03	0.18±0.03	0.28±0.04	0.14±0.03^b^	0.23±0.02^a^	0.8	0.96	0.02	0.79
MESF:PRF	2.50±0.12^a^	1.60±0.11^b^	1.50±0.19^b^	2.23±0.11^a^	1.62±0.40^b^	0.4	<0.0001	0.002	0.74
MESF:(MESF+PRF)	0.70±0.02^a^	0.60±0.01^b^	0.58±0.02^b^	0.68±0.01^a^	0.60±0.01^b^	0.19	0.0003	0.002	0.54

Data are presented as least square means±SEM. Effects within a row were significant at *P*<0.05 within the columns of postnatal nutrition or sex are marked by different superscripts. SUBF:PRF, ratios of subcutaneous to perirenal fat weights; MESF:PRF; ratios of mesenteric to perirenal fat weights; SUBF:MESF, ratios of subcutaneous to mesenteric fat weights; MESF:(MESF+PRF), ratios of mesenteric fat weights to the total weights of mesenteric and perirenal fats. For ED, LD, HCHF, CONV, EC, M and F see legends for Table 1.

There were no pre- or postnatal nutrition impacts on gross (except for pancreas) or relative (% of body weight) weights for any of the other studied (adipose) tissues or organs, although as reported earlier [4], kidney weights were reduced almost 20% in the 6-months old HCHF lambs compared to CONV lambs. A positive linear correlation was found between birth weight and adult gross pancreas weight (*P =* 0.0046)

### Body weight and proportions, fat deposition patterns and organ weights: sex impacts

Females generally had the lowest body weights, except for LOW, where females were heaviest, and HIGH males reached the highest body weights of all (S2 Table; *P* = 0.0003 and 0.0002 for ewe diet and sex interaction with or without EC, respectively).

Compared to males, female sheep had higher (*P<*0.05 for all unless otherwise stated, see Table 1 and S2 Table) BCS (3.2±0.05 vs. 2.8±0.05; *P<*0.0001), reduced size (% of body weight) of liver and m. *longissimus dorsii*, and increased contents (% of body weight; >2-fold higher) of all fats. This increase in fat content was more pronounced in SUBF (2.6-fold higher), PRF (2.4-fold higher) and sternal fat (2.5-fold higher) than MESF (1.8-fold) and pericardial fat (1.3-fold higher), which resulted in higher SUBF-to-MESF ratios (0.23 versus 0.14) and lower MESF-to-PRF ratios (1.62 vs 2.23) in females compared to males, and MESF constituted a lower proportion of intra-abdominal fat in females compared to males (*P* = 0.002; Table 2).

The impact of nutrition exposures in pre- and early postnatal life on relative weights of tissues or organs was identical in adult males and females, except for three tissues. Thus, LOW and NORM females had higher contents (% of body weight) of MESF and PRF fat than males, whereas the opposite sex impact was observed in HIGH sheep (*P =* 0.03 for ewe diet and sex interaction with EC; Table 1), and HCHF females had larger adrenals (% of body weight) than HCHF males (*P* = 0.03 for lamb diet and sex interaction with or without EC).

### Metabolic adaptations during fasting and tolerance tests: pre- and postnatal nutrition impacts

In this section, it will be described only for those parameters for which early nutrition impacts were markedly evident and other results are shown in (supplementary) figures. Noticeably, the LOW-HCHF sheep had higher plasma levels of BUN, cholesterol, creatinine, lactate and also lipid parameters (TG, NEFA) in one or more of the tolerance tests conducted as described below. Interestingly, these LOW-HCHF animals were also hypercholesterolaemic (ITT and PTT-fasted) and hyperureamic (PTT-fasted) as lambs at six months of age as reported elsewhere [5].

#### BUN

**Fig 1:**
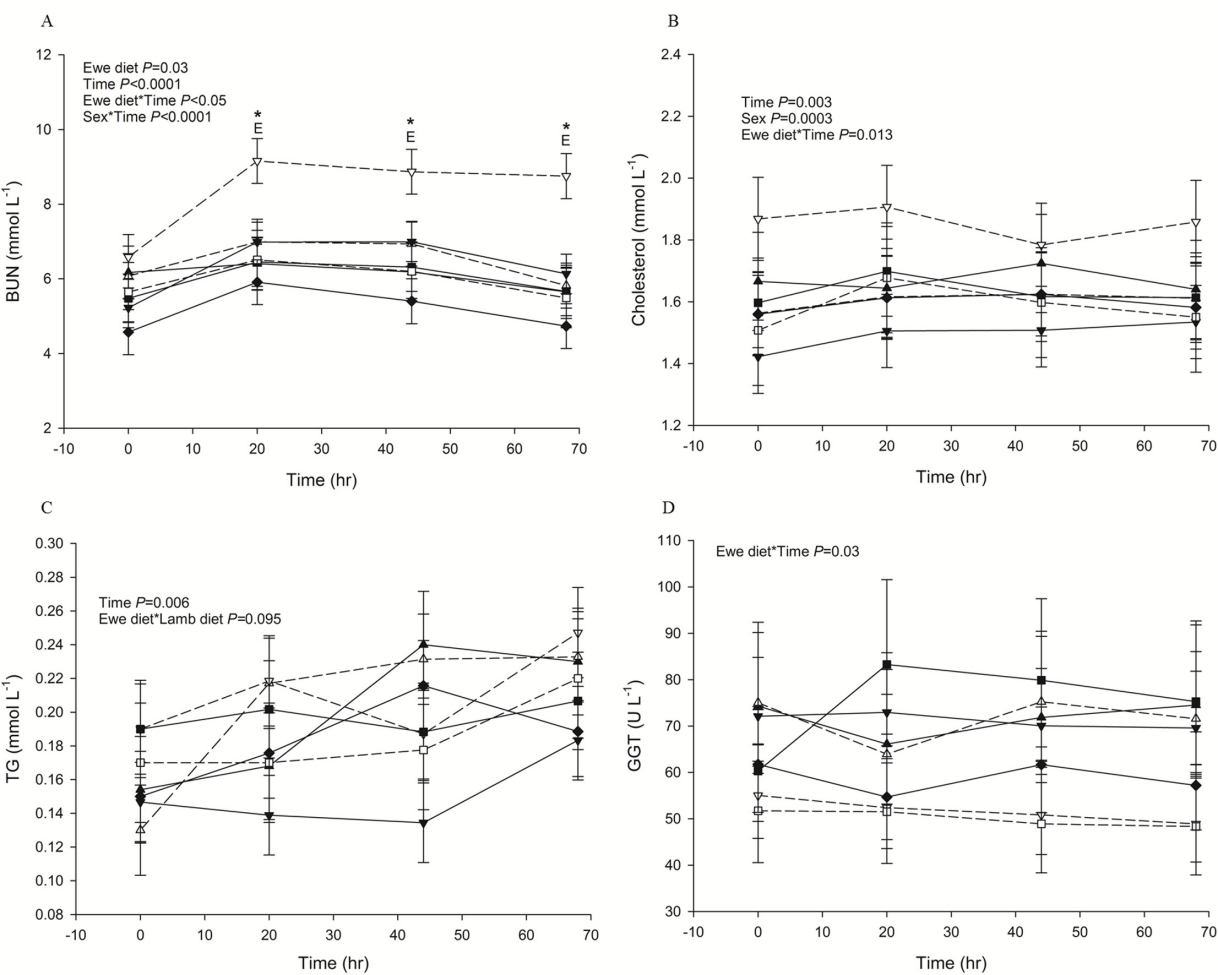
Changes in plasma metabolites during a 68-hour period of fasting. (A) BUN (blood urea nitrogen) (B) cholesterol (C) TG (triglycerides) and (D) GGT (γ-glutamyl transferase). The sheep were born to dams, which during the last 6 weeks of their twin-pregnancy were fed either a HIGH diet (fulfilling 150% of requirements for energy and 110% of requirements for protein) or a LOW diet (fulfilling only 50% of requirements for energy and protein) or a NORM diet (fulfilling 100% requirements for energy and protein). From 3 days after birth until 6 months of age (just after puberty), one twin was raised on an obesogenic high carbohydrate-high fat diet (HCHF; consisting of a cream-milk replacer mix in a 1:1 ratio supplemented with rolled maize) and the other twin was raised on a moderate hay-based diet (CONV; consisting of milk replacer and hay until 8 weeks and hay only thereafter and adjusted to achieve moderate and constant growth rates of approx. 225 g day ^-^1). Subgroups of sheep were slaughtered at 6 months of age, but the remaining sheep, which were used in the present study, were raised from 6 months of age until 2½ years of age (young adulthood) on a hay-based diet, resulting in 6 experimental groups: LOW-HCHF (N = 7; 4 males, 3 females; dash line, ▽); LOW-CONV (N = 9; 4 males, 5 females; solid line, ▼); HIGH-HCHF (N = 6; 3 males, 3 females; dash line, △); HIGH-CONV (N = 5; 2 males, 3 females; solid line, ▲); NORM-HCHF (N = 4; 2 males, 2 females; dash line, □); NORM-CONV (N = 6; 2 males, 4 females; solid line, ■). Age-matched control sheep from the same herd as the pregnant dams were purchased at 6 months of age and served as external controls (EC; N = 7: 3 males, 4 females; solid line, ♦). The values are presented as LS means ± SEM and the letters E and L denote pre- (**E**we diet) and postnatal diet (**L**amb diet) effects, respectively. The effects were significant at ***, *P*<0.001; **, *P*<0.01; *, *P*<0.05; #, *P<*0.1.

**Fig 2:**
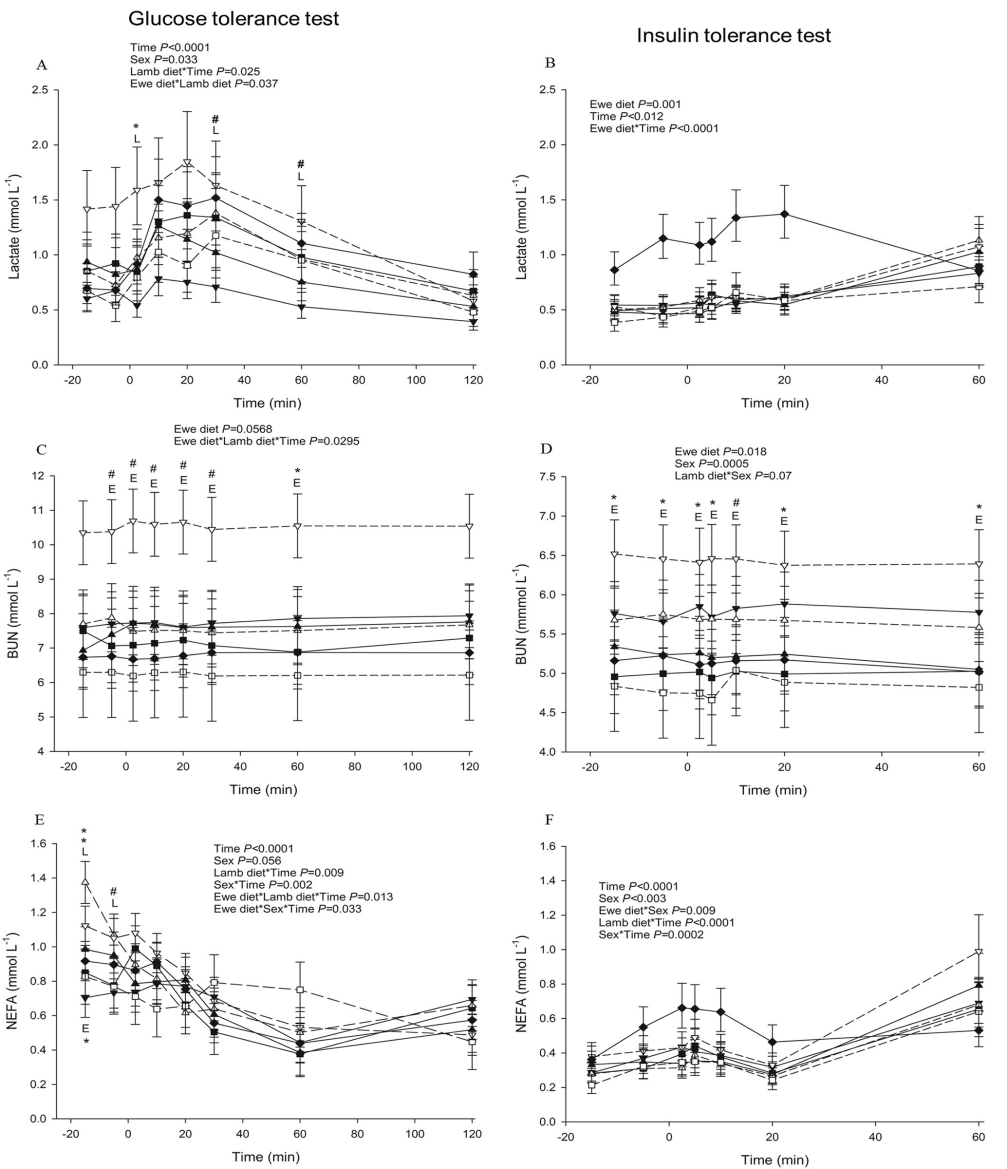
**Changes in blood metabolites during glucose (panels to the left; A, C, E) and insulin (panels to the right; B, D, F) tolerance tests.** (A) lactate (B) lactate (C) BUN (D) BUN (E) NEFA (non-esterified fatty acids) (F) NEFA. Data are shown for each combination of pre- and postnatal diet: LOW-HCHF (N = 7; 4 males, 3 females; dash line, ▽); LOW-CONV (N = 9; 4 males, 5 females; solid line, ▼); HIGH-HCHF (N = 6; 3 males, 3 females; dash line, △); HIGH-CONV (N = 5; 2 males, 3 females; solid line, ▲); NORM-HCHF (N = 4; 2 males, 2 females; dash line, □); NORM-CONV (N = 6; 2 males, 4 females; solid line, ■) and EC (N = 7: 3 males, 4 females; solid line, ♦). The values are presented as LS means ± SEM and the letters E and L denote pre- (**E**we diet) and postnatal diet (**L**amb diet) effects, respectively. The effects were significant at ***, *P*<0.001; **, *P*<0.01; *, *P*<0.05; #, *P<*0.1. HIGH, LOW, NORM, HCHF, CONV and EC: See legends to Fig 1.

LOW-HCHF sheep were consistently hyperuraemic during FTT, (*P* = 0.02 or <0.05 for the interaction of prenatal nutrition and time with or without EC; Fig 1A), GTT (NORM-HCHF were hypoureaemic; *P* = 0.029 or 0.095 for interaction of pre- and postnatal nutrition with or without EC; Fig 2C) and PTT (also in fasted but distinctly in fed state; *P =* 0.077 for interaction of pre- and postnatal nutrition with EC; S5B Fig) compared to the rest of the groups. Consistently higher BUN levels in LOW-HCHF sheep were also evident during ITT which led to increased BUN levels in LOW animals followed by HIGH, EC and NORM animals (*P* = 0.018 or *P* = 0.026 for prenatal nutrition with or without EC; Fig 2D).

#### Cholesterol

**Fig 3:**
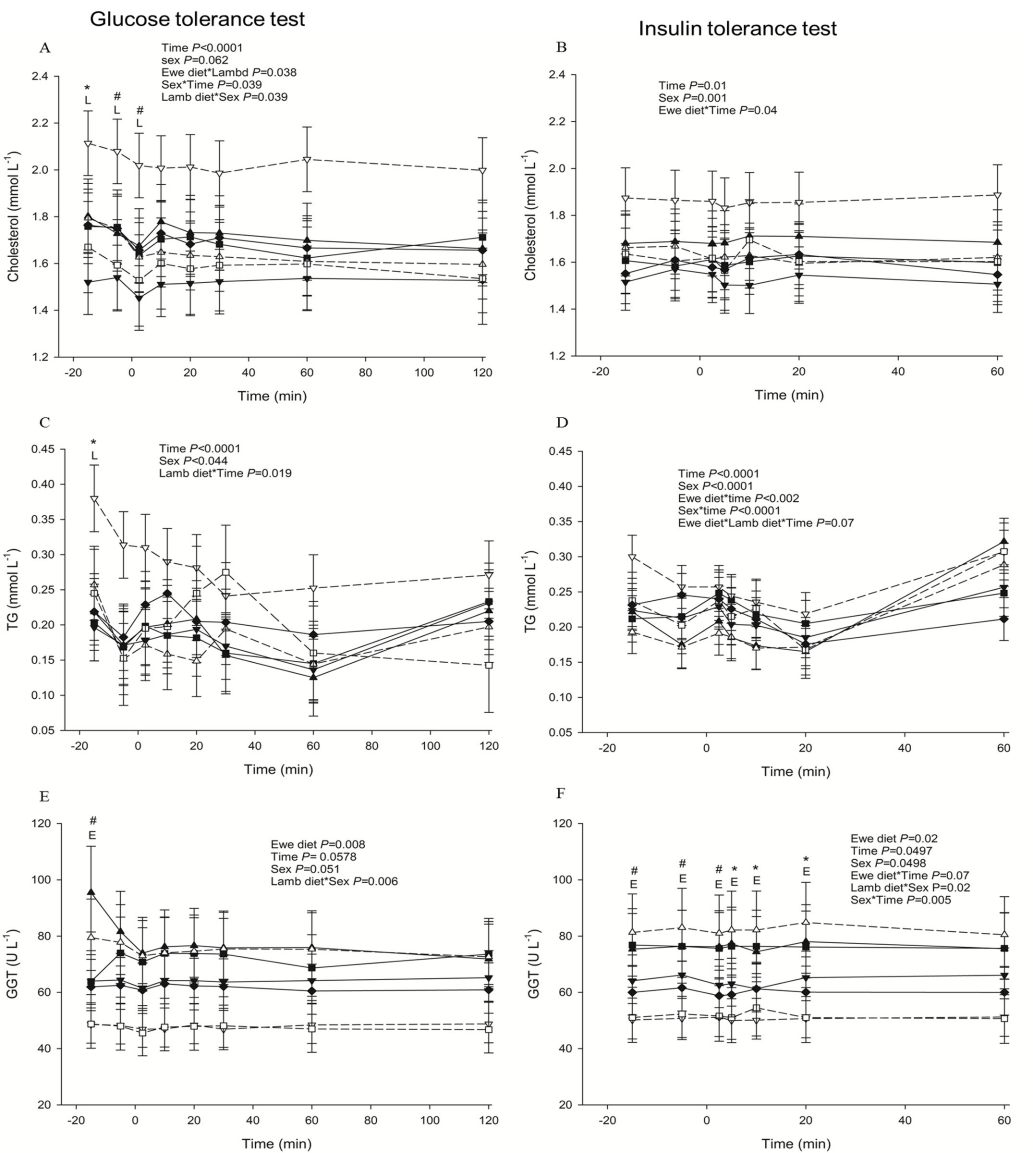
**Changes in plasma metabolites during glucose (panels to the left; A, C, E) and insulin (panels to the right; B, D, F) tolerance tests.** (A) cholesterol (B) cholesterol (C) TG (triglycerides) (D) TG (E) GGT (γ-glutamyl transferase) (F) GGT. Data are shown for each combination of pre- and postnatal diet: LOW-HCHF (N = 7; 4 males, 3 females; dash line, ▽); LOW-CONV (N = 9; 4 males, 5 females; solid line, ▼); HIGH-HCHF (N = 6; 3 males, 3 females; dash line, △); HIGH-CONV (N = 5; 2 males, 3 females; solid line, ▲); NORM-HCHF (N = 4; 2 males, 2 females; dash line, □); NORM-CONV (N = 6; 2 males, 4 females; solid line, ■) and EC (N = 7: 3 males, 4 females; solid line, ♦). The values are presented as LS means ± SEM and the letters E and L denote pre- (**E**we diet) and postnatal diet (**L**amb diet) effects, respectively. The effects were significant at ***, *P*<0.001; **, *P*<0.01; *, *P*<0.05; #, *P<*0.1. HIGH, LOW, NORM, HCHF, CONV and EC: See legends to Fig 1.

LOW-HCHF animals were also hypercholesterolaemic during FTT (*P =* 0.01 or 0.04 for interaction of prenatal nutrition and time with or without EC; Fig 1B), GTT (*P* = 0.038 or 0.04 for interaction of pre- and postnatal nutrition with or without EC; Fig 3A), ITT (*P =* 0.04 for interaction of prenatal nutrition and time with EC; Fig 3B) and PPT (also in fed but distinctly in fasted state; S5C Fig) and LOW-CONV sheep had generally lowest cholesterol levels in all these tests except PPT-fed.

#### Creatinine

**Fig 4:**
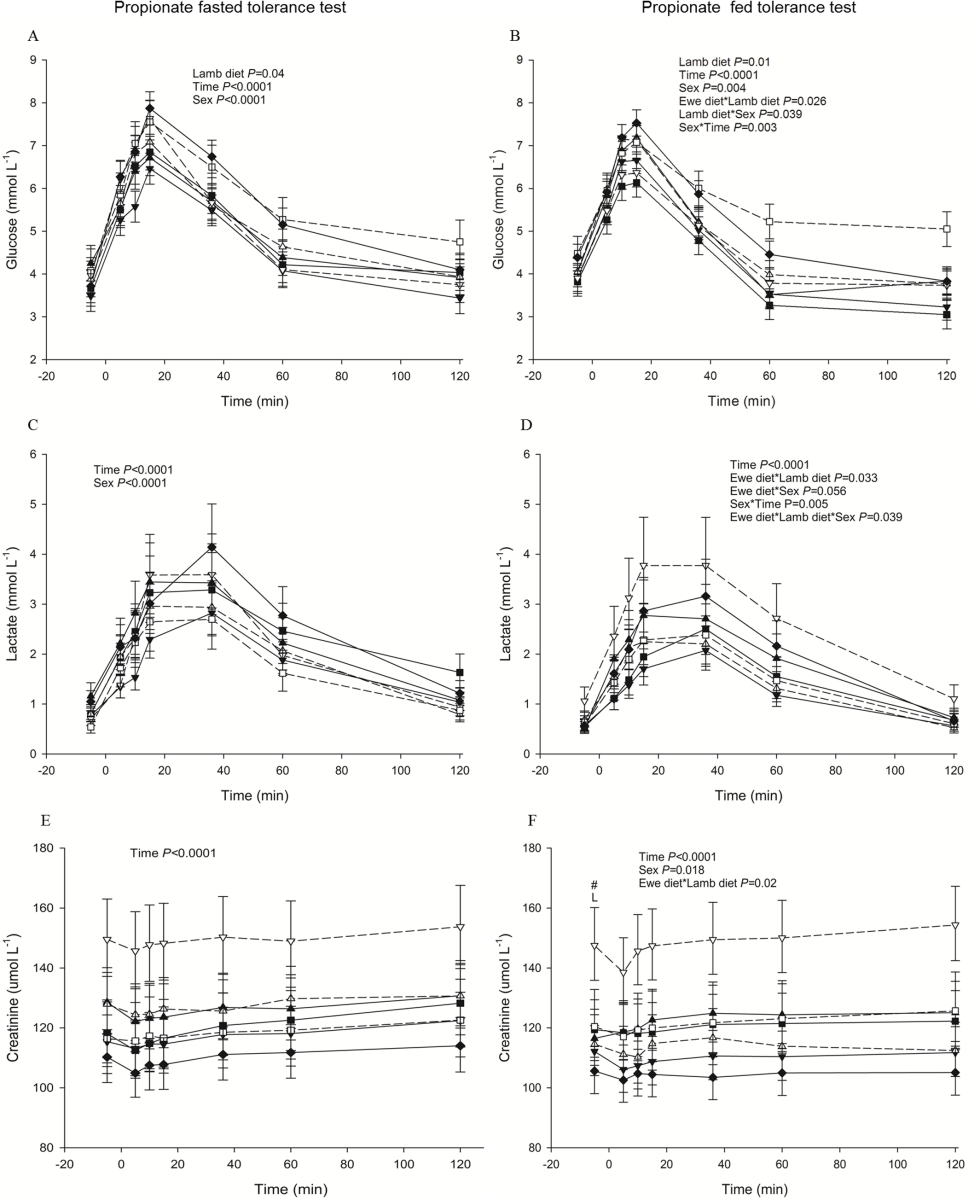
**Changes in plasma metabolites during a propionate tolerance test in 68-hour fasted (panels to the right; A, C, E) and fed (panels to the left; B, D, F) sheep.** (A) glucose (B) glucose (C) lactate (D) lactate (E) creatinine (F) creatinine. Data are shown for each combination of pre- and postnatal diet: LOW-HCHF (N = 7; 4 males, 3 females; dash line, ▽); LOW-CONV (N = 9; 4 males, 5 females; solid line, ▼); HIGH-HCHF (N = 6; 3 males, 3 females; dash line, △); HIGH-CONV (N = 5; 2 males, 3 females; solid line, ▲); NORM-HCHF (N = 4; 2 males, 2 females; dash line, □); NORM-CONV (N = 6; 2 males, 4 females; solid line, ■) and EC (N = 7: 3 males, 4 females; solid line, ♦). The values are presented as LS means ± SEM and the letters E and L denote pre- (**E**we diet) and postnatal diet (**L**amb diet) effects, respectively. The effects were significant at ***, *P*<0.001; **, *P*<0.01; *, *P*<0.05; #, *P<*0.1. HIGH, LOW, NORM, HCHF, CONV and EC: See legends to Fig 1.

LOW-HCHF animals were distinctly hypercreatinaemic during FTT (*P =* 0.005 for postnatal diet with EC; S2C Fig), GTT (*P =* 0.055 for interaction of pre- and postnatal nutrition without EC; S4C Fig), ITT (*P* = 0.03 for postnatal nutrition with EC; S4D Fig) and PTT-fed (*P* = 0.02 or 0.087 for interaction of pre- and postnatal nutrition with or without EC Fig 4F) than the rest of the groups and similar picture was also observed during PTT-fasted (Fig 4E).

#### Lactate

During GTT (except at 120 min; *P* = 0.037 or 0.044 for interaction of pre- and postnatal nutrition with or without EC; Fig 2A) and PPT-fed (*P =* 0.033 or 0.086 for interaction of pre- and postnatal nutrition with or without EC; Fig 4D), LOW-HCHF animals had the highest lactate levels and HIGH-CONV had the lowest lactate levels (more clearly during GTT) than the rest of the animals.

#### Glucose

During both PPT-fasted (*P* = 0.04 or 0.02 with or without EC; Fig 4A) and PPT-fed (*P =* 0.01 or 0.013 with EC or without EC: Fig 4B), HCHF animals had higher glucose levels than CONV animals due to generally highest glucose levels in NORM-HCHF sheep.

#### NEFA, TG and BOHB

**Fig 5:**
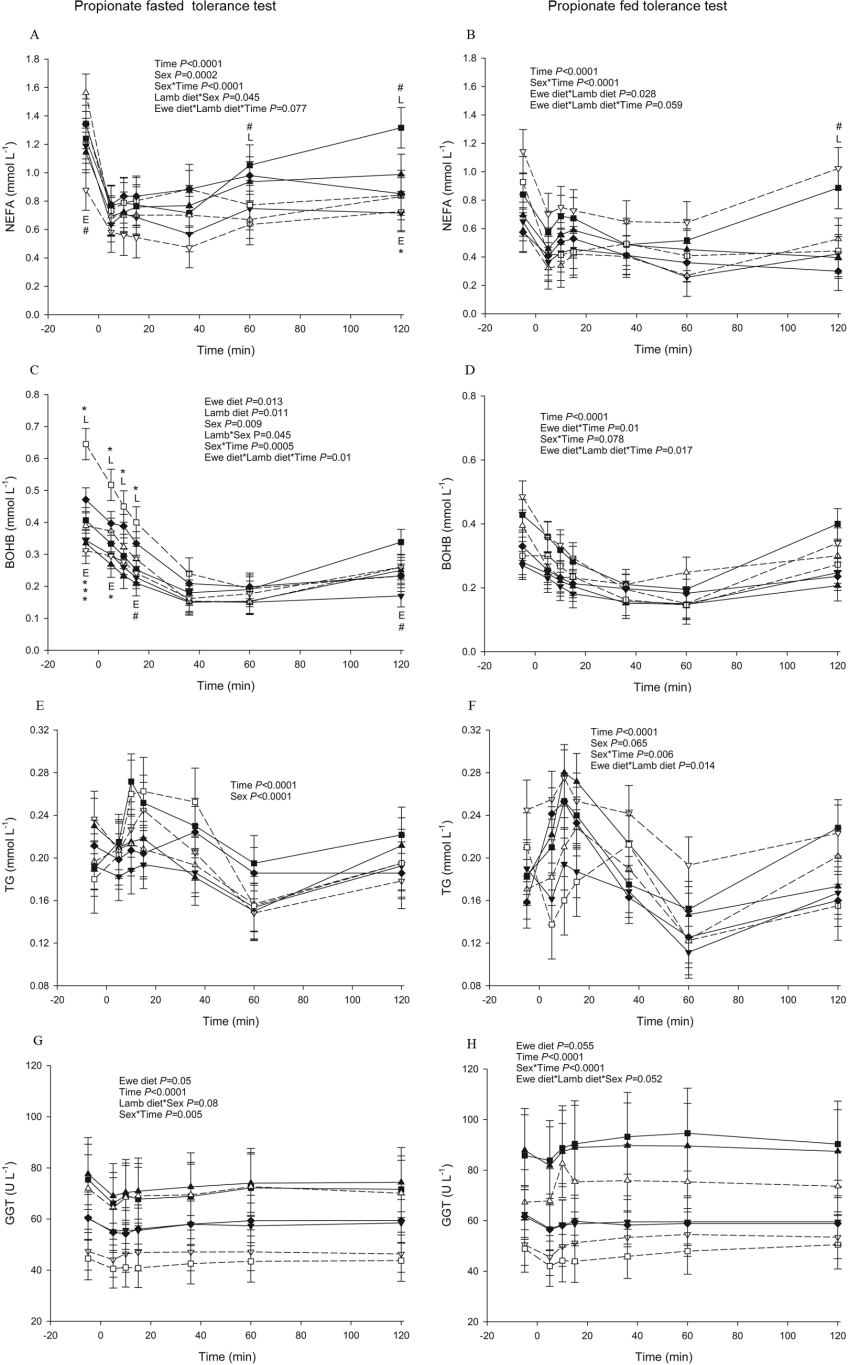
**Changes in plasma metabolites in sheep during propionate tolerance tests conducted after 68-hours of fasting (panels to left; A, C, E, G) and in the fed state (panels to the right; B, D, F, H) state.** (A) NEFA (non-esterified fatty acids) (B) NEFA (C) BOHB (β-hydroxy-butyrate) (D) BOHB (E) TG (triglycerides) (F) TG (G) GGT (γ-glutamyl transferase) (H) GGT. Data are shown for each combination of pre- and postnatal diet: LOW-HCHF (N = 7; 4 males, 3 females; dash line, ▽); LOW-CONV (N = 9; 4 males, 5 females; solid line, ▼); HIGH-HCHF (N = 6; 3 males, 3 females; dash line, △); HIGH-CONV (N = 5; 2 males, 3 females; solid line, ▲); NORM-HCHF (N = 4; 2 males, 2 females; dash line, □); NORM-CONV (N = 6; 2 males, 4 females; solid line, ■) and EC (N = 7: 3 males, 4 females; solid line, ♦). The values are presented as LS means ± SEM and the letters E and L denote pre- (**E**we diet) and postnatal diet (**L**amb diet) effects, respectively. The effects were significant at ***, *P*<0.001; **, *P*<0.01; *, *P*<0.05; #, *P<*0.1. HIGH, LOW, NORM, HCHF, CONV and EC: See legends to Fig 1.

NEFA, TG and BOHB responses were generally messy across the tolerance tests and hardly any conclusion could be drawn except that LOW-HCHF animals had visibly highest NEFA (*P =* 0.059 or 0.046 for pre- and postnatal nutrition and time interaction with or without EC; Fig 5B) and TG (except at 30 min post-injection; *P =* 0.019 for interaction of postnatal nutrition and time with EC; Fig 3C) levels during PPT-fed and GTT, respectively.

#### GGT

Overall HIGH animals had higher and LOW- and NORM-HCHF had consistently lowest GGT levels compared to the rest of the groups during FTT (*P =* 0.03 or 0.06 for the prenatal nutrition and time interaction with or without EC; Fig 1D), GTT (*P* = 0.008 with EC; *P =* 0.008 without EC; Fig 3E), ITT (particularly due to the highest levels in HIGH-HCHF sheep; *P* = 0.02 with EC; 3F), PPT-fasted (*P =* 0.05 with EC; Fig 5G) and PPT-fed (*P =* 0.055 with EC; Fig 5H).

### Metabolic adaptations during fasting and different tolerance tests: sex impacts

Impacts of sex were associated with differences in magnitude of responses, but in the same direction. Female sheep had higher plasma levels of lactate (GTT, PTT-fast), BUN (ITT), creatinine (ITT), cholesterol (in all except GTT), TG (in all tests), NEFA (GTT, PTT-fast) glucose (in all except ITT), BOHB (GTT, PTT-fast) and GGT (ITT, PTT-fast and -fed) than in males (*P<*0.05 for all with or without EC). **During GTT**, GGT (*P =* 0.006 for interaction of postnatal diet and sex with EC) and cholesterol (*P* = 0.039 for interaction of postnatal diet and sex with EC) levels were higher in female animals than males particularly in the HCHF-fed animals and NEFA levels higher in particularly LOW-females than LOW-males (*P =* 0.0328 for prenatal nutrition, sex and time interaction with EC). **During ITT**, females animals had higher NEFA (in HIGH and NORM but not in LOW; *P* = 0.001 for prenatal nutrition and sex with EC) and BOHB levels (less pronounced in HCHF than CONV; *P* = 0.011 for postnatal diet and sex interaction with EC) than males. **During PPT-fed**, creatinine and lactate (particularly evident in LOW-HCHF animals; *P =* 0.039 for interaction of pre- and postnatal nutrition and sex with EC) levels were higher in male than in female animals.

## Discussion

This study confirmed that under- and overnutrition in late foetal life have differential implications for metabolic adaptability in adulthood, which predispose foetally undernourished individuals for development of adverse metabolic traits upon early postnatal obesity development, and we have shown that this cannot be compensated by dietary correction later in life. Our studies also revealed that early postnatal obesity development prevents expansion or interferes with ability of PRF to expand later in life, which to a higher extent will direct abdominal fat deposition in direction of MESF. This was, however, not directly linked to adverse metabolic outcomes except in LOW sheep. Differential responses in males and females to malnutrition in late foetal life (body weight, MESF and PRF) or an obesogenic diet in early postnatal life (adrenals) were observed for a very few parameters. As mentioned earlier since there was no even distribution of sexes across the experimental groups in this study, sex issues will therefore not be given much attention in the following.

### Obesity development in early life has long-term implications for perirenal fat expandability and thereby intra-abdominal fat distribution patterns

Malnutrition in prenatal life and obesity development in early postnatal life (followed by body fat correction later on) were exclusively altering body contents of PRF and MESF, except for a sex specific response to early obesity development in adrenals.

A major and most surprising finding was the complete absence of any expansion of PRF from adolescence [4] into adulthood in the sheep, which became obese in early life and subsequently lost body fat after puberty in response to a dietary correction. All other organs and (adipose) tissues were increased in gross weights from adolescence to adulthood. The lack of expansion of PRF was associated with an altered distribution of intra-abdominal fat in adults. Thus, in HCHF sheep, MESF constituted 10%-unit more of the intra-abdominal fat compared to CONV sheep, and this despite the fact that the previously obese adult HCHF sheep had been fed a moderate and body fat correcting diet for 2 years, and achieved the same overall body weights as CONV sheep, which had been fed a moderate diet throughout postnatal life. Exposure to late gestation undernutrition pulled in the same direction (but much less dramatic) as early obesity to reduce fat deposition in PRF in adult sheep, whereas no prenatal nutrition impacts were observed in any of the other adipose tissues.

Previous work relating to foetal programming and obesity development to metabolic disorders later in life, have pointed to adipose tissue expandability, particularly in SUBF, as a major risk factor for development of visceral obesity and associated metabolic disorders, and SUBF is believed to be a healthier fat than MESF [17, 18]. We have in this [4] and a previous sheep experiment [16] found that prenatal undernutrition can reduce expandability of SUBF, which appears to predispose for abdominal fat deposition during the development of obesity. However, the results presented in this paper somewhat surprisingly showed that when obese HCHF lambs were transferred to the same moderate diet as that fed to CONV lambs, then they ended up having the same SUBF content 2 years later as adults, due to a massive increase in SUBF development in CONV sheep (10-fold) and a continued expansion (2-fold) in proportion to the general development in body weight in HCHF sheep from adolescence into adulthood.

This does not fit well with the theory that reduced expandability in SUBF should be a major explanatory factor for the preference for storage of fat in MESF in the adult HCHF sheep. Thus at least after the HCHF sheep stopped being exposed to an obesogenic diet, SUBF were capable of continued expansion to even out differences across all experimental groups. Thus, our study rather points to a long-term impact of early obesity development, which renders PRF unable to continue to expand in previously obese subjects. We have not been able to find any explanation for this tissue specific behaviour.

It is well established that visceral adiposity is a risk factor for development of metabolic disorders such as the metabolic syndrome and type-2 diabetes in humans. Gabriely et al. [19] elegantly showed that surgical removal of visceral, i.e. epididymal and perinephric fat at 20-months of age in two strain of rats could restore insulin action to that observed in young rats and delay the onset of diabetes in an intrinsically diabetic rat strain. However, the specific contributions of the individual intra-abdominal adipose tissues (omental, mesenteric versus perirenal) in this context are not known. Such studies in humans are complicated by the anatomical position of this tissue deep in the abdominal cavity, and the intraperitoneal and retroperitoneal fat masses are not easily distinguishable in CT scans and MRI [20].

As adolescents, the HCHF fed lambs had significantly higher depositions of fat in all the studied fat depots, but the fat accumulation was particularly extensive in PRF [4], and the PRF adipocyte size in the obese lambs was almost 1.8 and 1.4-fold higher than observed in SUBF and MESF, respectively (P. Khanal et al., unpublished results). Sethi and Vidal-Puig [21] have introduced the idea of adipose tissue expandability and a maximal fixed capacity for safely storing of fat in a tissue. According to this idea, fat will be deposited elsewhere, when this capacity is exceeded, and our results indicate that the PRF capacity may be significantly affected by the nutrition history in both prenatal and particularly early postnatal life. Our results further indicate that early obesity-induced damage in PRF cannot be rescued later in life upon dietary correction, whereas this appears to be possible in SUBF, in which adipocytes are not capable of undergoing hyperplasia during obesity development to the same extent as in PRF.

It was noteworthy that kidney size was similar in adult HCHF and CONV sheep, since the HCHF diet reduced kidney size in lambs by 20% in this [4] and a previous sheep experiment [16]. Kidney function and development may be adversely affected by excessive fat deposition in PRF due to compression of renal vessels and parenchyma, which will affect renal blood flow and tubular flow rate [22, 23] explaining the reduced kidney size in lambs. However, the kidney must apparently be able to exhibit compensatory growth from adolescence into adulthood, provided a situation is created, where relative fat mass of PRF is decreased. This may have long-term implications for development of renal disorders, although we did find indications, as discussed in the following, to suggest that the early obesity development may have long-term adverse implications for kidney function independently of kidney weight.

### Exposure to under- and overnutrition in late foetal life have differential long-term implications for metabolic adaptability in adult life

We have previously shown that the LOW lambs included in this study were predisposed already at 6-months of age for development of hypercholesterolaemia and hyperuraemia upon feeding the obesogenic HCHF diet during the first 6-months of postnatal life, whereas HIGH lambs became hyperglycaemic compared to other groups upon exposure to the HCHF diet [5]. In the adult HIGH-HCHF sheep, there were no longer any signs of hyperglycaemia, after the sheep had been fed a moderate diet for two years. However, the fetal predisposition for hypercholesterolaemia and hyperuraemia, observed in the LOW lambs fed the obesogenic HCHF diet [5] persisted into adulthood, and not even after 2 years on a moderate diet were these traits reversed in the LOW-HCHF sheep. Liver plays an important role in the cholesterol homeostasis [24] and studies also in humans have demonstrated that impaired growth during late gestation is associated with raised blood cholesterol levels in adult life, and altered liver growth has been suggested as a responsible factor for such permanent alterations in cholesterol metabolism [25]. Detailed information is missing with regards to what alterations in cholesterol metabolism in the liver or other tissues are responsible for the sustained elevations of cholesterol levels throughout early and adult life in prenatally undernourished individuals. However, previous study in rats demonstrates that maternal protein restriction during pregnancy and lactation increased circulating and hepatic cholesterol levels in adult offspring due to repressive epigenetic alternations at the promoter of hepatic cholesterol 7α-hydroxylase gene [26]. Moreover exposure to low protein diet during pregnancy created hypercholesterolaemia in ApoE*3-Leiden mice offspring, which co-existed with development of severe atherosclerotic lesions within the aortic arch and suppressed mRNA expression of LDL receptor-related protein 1 and hepatic sterol regulatory element-binding protein-1c genes [27]. The extent of such dyslipidaemia was further increased upon exposure to a postnatal atherogenic diet. These previous studies support our findings that prenatal undernutrition leads to long-term implications for cholesterol homeostasis and exposure to an obesogenic diet early in life can worsen the consequences irreversibly.

The LOW-HCHF sheep in addition to hypercholesterolaemia also developed hyperuraemia, hypercreatinaemia and hyperlactaemia, of which the two first were indicators of alterations in hepatic and/or renal function. BUN itself has been considered a marker, although not very specific, for renal function as it is determined by the balance between hepatic urea production and excretion by kidneys [28]. There were slight indications of hyperureamia in HCHF sheep already at 6-months of age during exposure to PTT in the fasted state [5] and their kidney size was as already mentioned significantly reduced [4]. It has also been shown in adult rats that offspring exposed to maternal protein restriction *in utero* had higher BUN levels, and interestingly creatinine clearance was also significantly reduced in these offspring, when they were young, along with elevated blood pressure at all ages [29]. Serum (and hence also plasma) creatinine is a commonly used as a rough marker of renal function, and serum creatinine levels will rise in response to poor clearance of creatinine by kidneys [30], although other extra-renal factors also affect creatinine levels. Creatinine is produced from muscle creatine phosphate and at a fairly constant rate depending on muscle mass [31]. We did not, however, find any indications of differences in muscle mass depending on the prenatal nutrition history in this or our previous sheep study [16]. Previous studies in humans and animals have revealed a strong association between intrauterine growth restriction and renal dysfunction later in life [32–35]. This study indicates that growth restriction of kidneys in HCHF-fed lambs could be recovered after dietary correction from adolescence to adulthood, however, renal function in prenatally undernourished animals appear to be permanently affected, possibly due to a reduction in nephron number as observed in intrauterine growth restricted new born piglets [36]. Future studies are obviously needed to confirm that development of hyperuraemia and hypercreatinaemia in LOW-HCHF sheep observed in this study were caused by disturbance of renal function. However, alterations in cholesterol, creatinine and BUN metabolism in the LOW-HCHF sheep indicate that liver and kidney may be the key target organs responsible for long-term programming of metabolic adaptability in response to late gestation undernutrition, placing such individuals at risk of irreversible damage in response to early obesity development. Individuals exposed to late gestation overnutrition, however, appear to be reversible upon metabolic features upon dietary intervention to correct early obesity development.

Our studies confirm those of others [37–39] that prenatal nutrition implications in adults are expressed in both sexes. We did not find convincing evidence to suggest that female animals should be better protected towards adverse impacts of foetal programming, as others have suggested [40–42]. The only findings pointing in that direction were higher lactate and creatinine responses in LOW-HCHF males in PTT-fed, and females may tend to deposit relatively more fat in PRF and SUBF than MESF compared to males (ratio between females-to-males with respect to fat mass were 2.2, 2.5 and 1.6, respectively, in the three tissues based on values from Table 1). Pulling in the other direction were the findings that the adult female sheep had higher fat deposition in all adipose tissues studied, more pronounced NEFA responses to glucose injection, and HCHF females had higher adrenals weight and increased GGT and cholesterol levels during GTT compared to males. The quantitative sex specific differences in gross weights of other tissues and organs depending on the prenatal nutrition history all disappeared when expressed as a percentage of body weight.

## Conclusions

In conclusion, our study has shown that late gestation undernutrition predisposed for adult hypercholesterolaemia, hyperureamia, hypercreatinaemia and hyperlactataemia, but these adverse metabolic traits only became manifested when the sheep in addition had been exposed to a mismatching obesogenic diet in early postnatal life. Sheep with a history of late gestation overnutrition, however, were resistant towards such adverse long-term impacts of obesity development during adolescence, since they attained normal metabolic adaptability in adulthood upon normalization of their diet for 2 years. Early obesity development was associated with a complete lack of expansion of PRF but not of other adipose tissues from adolescence to adulthood, resulting in MESF constituting a greater proportion of intra-abdominal fat. Prenatal undernutrition had a similar but much less pronounced effect. This was not associated with adverse metabolic features *per se* in adulthood, except in individuals with a history of mismatching foetal undernutrition. It may thus be appropriate to consider differential nutritional strategies for individuals born at different extremes of the birth weight spectrum. Both males and females manifest these long-term consequences of pre- and early postnatal nutrition.

## Supporting Information

S1 Fig Changes in body weights of sheep from 8 months until 2 years of age. The sheep were born to dams, which during the last 6 weeks of their twin-pregnancy were fed either a HIGH diet (fulfilling 150% of requirements for energy and 110% of requirements for protein) or a LOW diet (fulfilling only 50% of requirements for energy and protein) or a NORM diet (fulfilling 100% requirements for energy and protein). From 3 days after birth until 6 months of age (just after puberty), one twin was raised on an obesogenic high-carbohydrate-high fat diet (HCHF; consisting of a cream-milk replacer mix in a 1:1 ratio and supplemented with rolled maize) and the other twin was raised on a moderate hay-based diet (CONV; consisting of milk replacer and hay until 8 weeks and hay only thereafter and adjusted to achieve moderate and constant growth rates of approx. 225 g day ^-^1). Subgroups of sheep were slaughtered at 6 months of age, but the remaining sheep, which were used in the present study, were raised from 6 months of age until 2½ years of age (young adulthood) on a hay-based diet supplemented during the first months with rolled barley and a commercial concentrate in amounts fulfilling requirements for moderate growth rates initially and maintenance as adulthood was reached. This resulted in 6 experimental groups: LOW-HCHF (N = 7; 4 males, 3 females; dash line, ▽); LOW-CONV (N = 9; 4 males, 5 females; solid line, ▼); HIGH-HCHF (N = 6; 3 males, 3 females; dash line, △); HIGH-CONV (N = 5; 2 males, 3 females; solid line, ▲); NORM-HCHF (N = 4; 2 males, 2 females; dash line, □); NORM-CONV (N = 6; 2 males, 4 females; solid line, ■). Age-matched control sheep from the same herd as the pregnant dams were purchased at 6 months of age and served as external controls (EC; N = 7: 3 males, 4 females; solid line, ♦). Values are presented as LS means with SEM represented by vertical bars. Within week, LS means were significantly different at *P*<0.05.
(TIF)

S2 Fig Changes in plasma metabolites during a 68-hour period of fasting. (A) glucose (B) lactate (C) creatinine (D) NEFA (non-esterified fatty acids) (E) BOHB (β-hydroxy-butyrate). Data are shown for each combination of pre- and postnatal diet: LOW-HCHF (N = 7; 4 males, 3 females; dash line, ▽); LOW-CONV (N = 9; 4 males, 5 females; solid line, ▼); HIGH-HCHF (N = 6; 3 males, 3 females; dash line, △); HIGH-CONV (N = 5; 2 males, 3 females; solid line, ▲); NORM-HCHF (N = 4; 2 males, 2 females; dash line, □); NORM-CONV (N = 6; 2 males, 4 females; solid line, ■) and EC (N = 7: 3 males, 4 females; solid line, ♦). HIGH, LOW, NORM, HCHF, CONV and EC: See legends to S1 Fig.
(TIF)

S3 Fig Area under curves. (A) Area under the curve (AUC) for plasma glucose (AUC glucose; glucose tolerance test) (B) Area over the curve (AOC) for plasma glucose (insulin tolerance test) (C) AUC for plasma glucose (propionate tolerance test after 68 hours of fasting) (D) AUC for plasma glucose (propionate tolerance test in the fed state). Data are shown for each combination of pre- and postnatal diet: LOW-HCHF (N = 7; 4 males, 3 females); LOW-CONV (N = 9; 4 males, 5 females); HIGH-HCHF (N = 6; 3 males, 3 females); HIGH-CONV (N = 5; 2 males, 3 females); NORM-HCHF (N = 4; 2 males, 2 females); NORM-CONV (N = 6; 2 males, 4 females) and EC (N = 7: 3 males, 4 females). HIGH, LOW, NORM, HCHF, CONV and EC: See legends to S1 Fig.
(TIF)

S4 Fig **Changes in plasma metabolites during glucose (panels to the left; A, C, E) and insulin (panels to the right; B, D, F) tolerance tests**. (A) glucose (B) glucose (C) creatinine (D) creatinine (E) BOHB (β-hydroxy-butyrate) (F) BOHB. Data are shown for each combination of pre- and postnatal diet: LOW-HCHF (N = 7; 4 males, 3 females; dash line, ▽); LOW-CONV (N = 9; 4 males, 5 females; solid line, ▼); HIGH-HCHF (N = 6; 3 males, 3 females; dash line, △); HIGH-CONV (N = 5; 2 males, 3 females; solid line, ▲); NORM-HCHF (N = 4; 2 males, 2 females; dash line, □); NORM-CONV (N = 6; 2 males, 4 females; solid line, ■) and EC (N = 7: 3 males, 4 females; solid line, ♦). HIGH, LOW, NORM, HCHF, CONV and EC: See legends to S1 Fig.
(TIF)

S5 Fig **Changes in plasma metabolites during a propionate tolerance test in 68-hour fasted (panels to the left; A, C) and fed (panels to the right; B, D) sheep.** (A) BUN (blood urea nitrogen) (B) BUN (C) cholesterol (D) cholesterol. Data are shown for each combination of pre- and postnatal diet: LOW-HCHF (N = 7; 4 males, 3 females; dash line, ▽); LOW-CONV (N = 9; 4 males, 5 females; solid line, ▼); HIGH-HCHF (N = 6; 3 males, 3 females; dash line, △); HIGH-CONV (N = 5; 2 males, 3 females; solid line, ▲); NORM-HCHF (N = 4; 2 males, 2 females; dash line, □); NORM-CONV (N = 6; 2 males, 4 females; solid line, ■) and EC (N = 7: 3 males, 4 females; solid line, ♦). HIGH, LOW, NORM, HCHF, CONV and EC: See legends to S1 Fig.
(TIF)

S1 Table Changes in fat deposition pattern from 6 months of age (adolescence) until 2½ years of age (adulthood) during which period all animals were fed a moderate diet. Data are presented as least square means±SEM for organ and tissue weights or expressed as percentage of body weight at 2½ years of age. Fold increase in weights of organs and tissues at 2½ years as compared to 6 months of age has been calculated based on the organ and tissue data from 6 months old lambs which were published previously (Khanal, et al., 2014). NORM (N = 10; 4 males, 6 females), normal diet fulfilling requirements for energy and protein; HIGH (N = 11; 5 males, 6 females), 150% of requirements for energy and 110% of requirements for protein; LOW (N = 16; 8 males, 8 females), 50% of requirements for energy and protein; HCHF (n = 13; 8 males, 5 females), high carbohydrate-high fat diet from birth until six months of age and hay-based normal diet thereafter until 2½ years of age; CONV (N = 13; 7 males, 6 females) conventional diet to achieve moderate and constant growth rates of appr. 225 g day-1 from birth until six months of age and hay-based normal diet thereafter until 2½ years of age; EC, external controls (N = 7; 3 males, 4 females). NORM-CONV (N = 6; 2 males, 4 females); NORM-HCHF (N = 4; 2 males, 2 females); HIGH-CONV (N = 5; 2 males, 3 females); HIGH-HCHF (N = 6; 3 males, 3 females); LOW-CONV (N = 9; 4 males, 5 females); LOW-HCHF (N = 7; 4 males, 3 females); EC (N = 7; 3 males, 4 females).
(DOCX)

S2 Table Interactive effects of ewe diet and sex on different organ and tissue weights in 2½ year old sheep. Data are presented as least square means±SEM. Values within a row marked by different superscripts are significantly different at P<0.05. For NORM, HIGH and LOW see legends for S1 Table; EC, external controls (N = 7; 3 males, 4 females); M, males; F, females.
(DOCX)
