# Supplementary figures and images for: Long-Term Impacts of Foetal Malnutrition Followed by Early Postnatal Obesity on Fat Distribution Pattern and Metabolic Adaptability in Adult Sheep
Source: PLoS One. 2016 Jun 3;11(6):e0156700. doi: 10.1371/journal.pone.0156700 (PMC4892656; doi:10.1371/journal.pone.0156700)

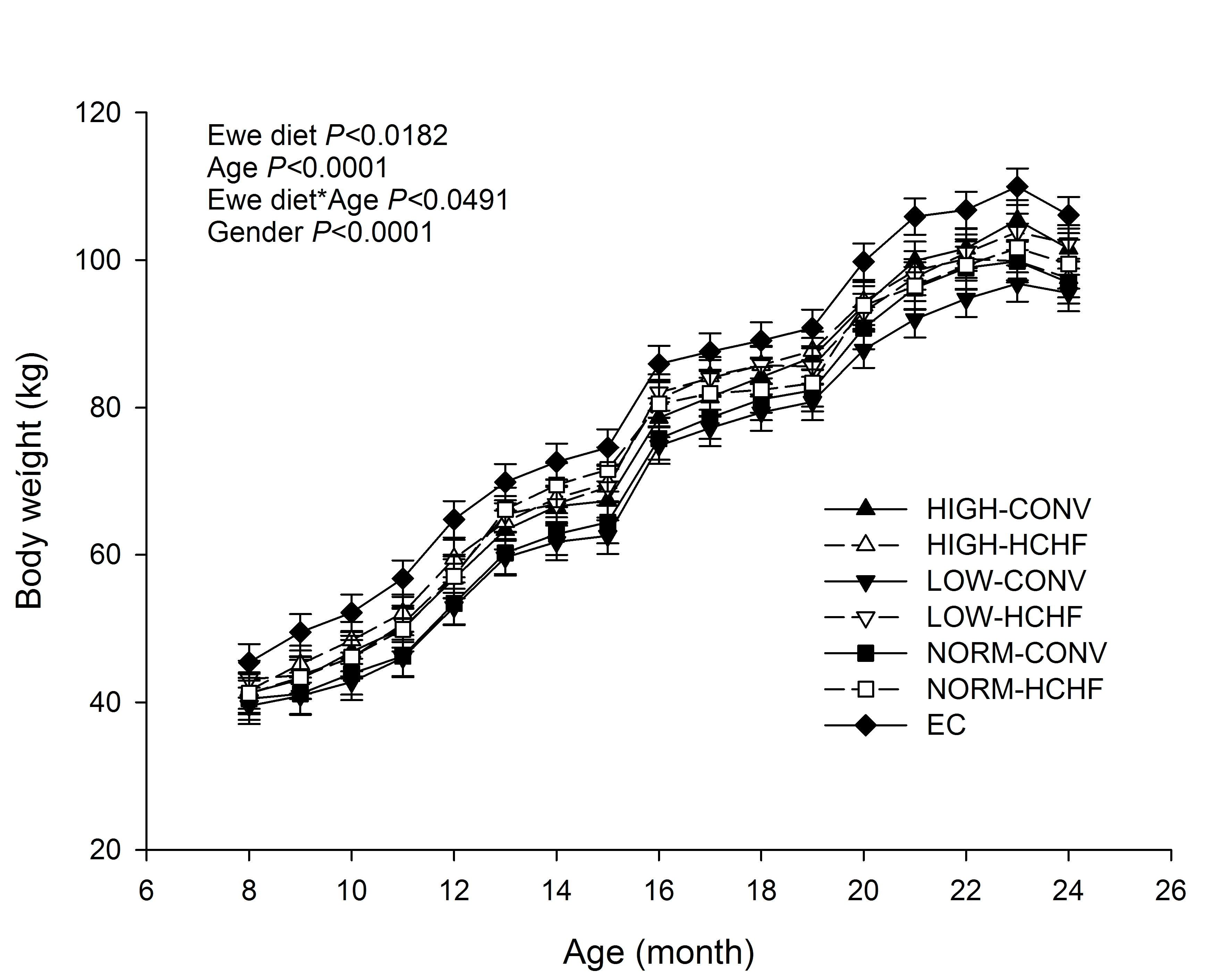

Supplement: S1 Fig — The sheep were born to dams, which during the last 6 weeks of their twin-pregnancy were fed either a HIGH diet (fulfilling 150% of requirements for energy and 110% of requirements for protein) or a LOW diet (fulfilling only 50% of requirements for energy and protein) or a NORM diet (fulfilling 100% requirements for energy and protein). From 3 days after birth until 6 months of age (just after puberty), one twin was raised on an obesogenic high-carbohydrate-high fat diet (HCHF; consisting of a cream-milk replacer mix in a 1:1 ratio and supplemented with rolled maize) and the other twin was raised on a moderate hay-based diet (CONV; consisting of milk replacer and hay until 8 weeks and hay only thereafter and adjusted to achieve moderate and constant growth rates of approx. 225 g day -1). Subgroups of sheep were slaughtered at 6 months of age, but the remaining sheep, which were used in the present study, were raised from 6 months of age until 2½ years of age (young adulthood) on a hay-based diet supplemented during the first months with rolled barley and a commercial concentrate in amounts fulfilling requirements for moderate growth rates initially and maintenance as adulthood was reached. This resulted in 6 experimental groups: LOW-HCHF (N = 7; 4 males, 3 females; dash line, ▽); LOW-CONV (N = 9; 4 males, 5 females; solid line, ▼); HIGH-HCHF (N = 6; 3 males, 3 females; dash line, △); HIGH-CONV (N = 5; 2 males, 3 females; solid line, ▲); NORM-HCHF (N = 4; 2 males, 2 females; dash line, □); NORM-CONV (N = 6; 2 males, 4 females; solid line, ■). Age-matched control sheep from the same herd as the pregnant dams were purchased at 6 months of age and served as external controls (EC; N = 7: 3 males, 4 females; solid line, ♦). Values are presented as LS means with SEM represented by vertical bars. Within week, LS means were significantly different at P<0.05. (TIF) [file pone.0156700.s001.TIF]

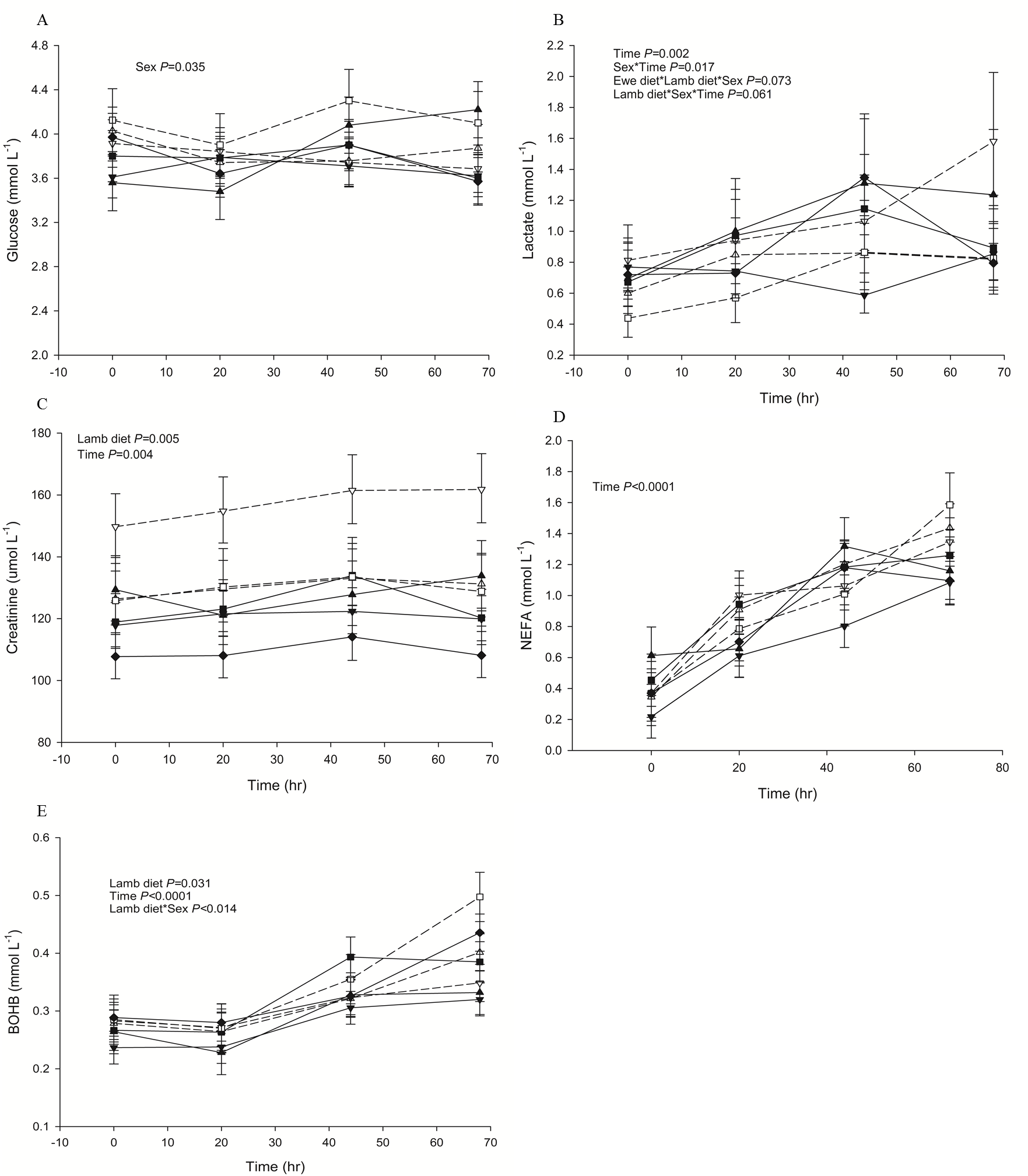

Supplement: S2 Fig — (A) glucose (B) lactate (C) creatinine (D) NEFA (non-esterified fatty acids) (E) BOHB (β-hydroxy-butyrate). Data are shown for each combination of pre- and postnatal diet: LOW-HCHF (N = 7; 4 males, 3 females; dash line, ▽); LOW-CONV (N = 9; 4 males, 5 females; solid line, ▼); HIGH-HCHF (N = 6; 3 males, 3 females; dash line, △); HIGH-CONV (N = 5; 2 males, 3 females; solid line, ▲); NORM-HCHF (N = 4; 2 males, 2 females; dash line, □); NORM-CONV (N = 6; 2 males, 4 females; solid line, ■) and EC (N = 7: 3 males, 4 females; solid line, ♦). HIGH, LOW, NORM, HCHF, CONV and EC: See legends to S1 Fig. (TIF) [file pone.0156700.s002.tif]

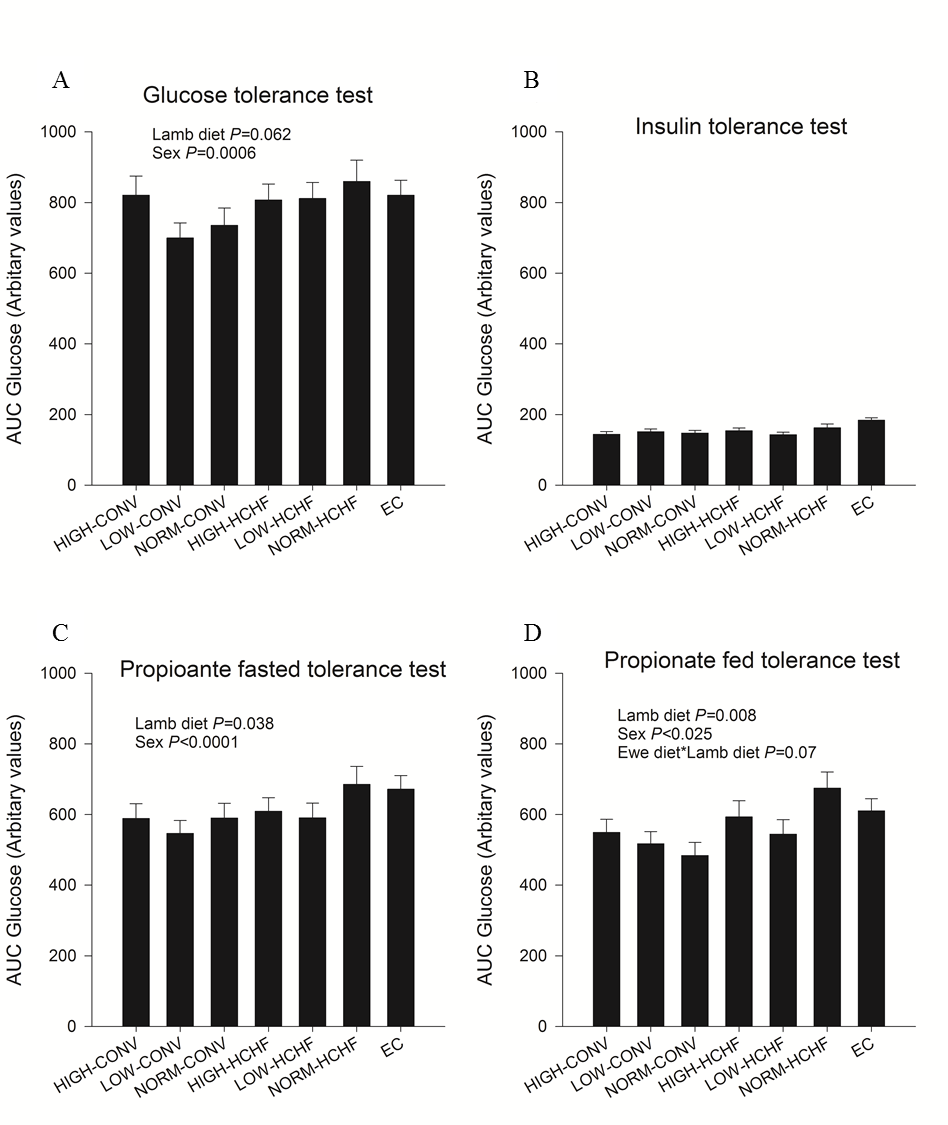

Supplement: S3 Fig — (A) Area under the curve (AUC) for plasma glucose (AUC glucose; glucose tolerance test) (B) Area over the curve (AOC) for plasma glucose (insulin tolerance test) (C) AUC for plasma glucose (propionate tolerance test after 68 hours of fasting) (D) AUC for plasma glucose (propionate tolerance test in the fed state). Data are shown for each combination of pre- and postnatal diet: LOW-HCHF (N = 7; 4 males, 3 females); LOW-CONV (N = 9; 4 males, 5 females); HIGH-HCHF (N = 6; 3 males, 3 females); HIGH-CONV (N = 5; 2 males, 3 females); NORM-HCHF (N = 4; 2 males, 2 females); NORM-CONV (N = 6; 2 males, 4 females) and EC (N = 7: 3 males, 4 females). HIGH, LOW, NORM, HCHF, CONV and EC: See legends to S1 Fig. (TIF) [file pone.0156700.s003.tif]

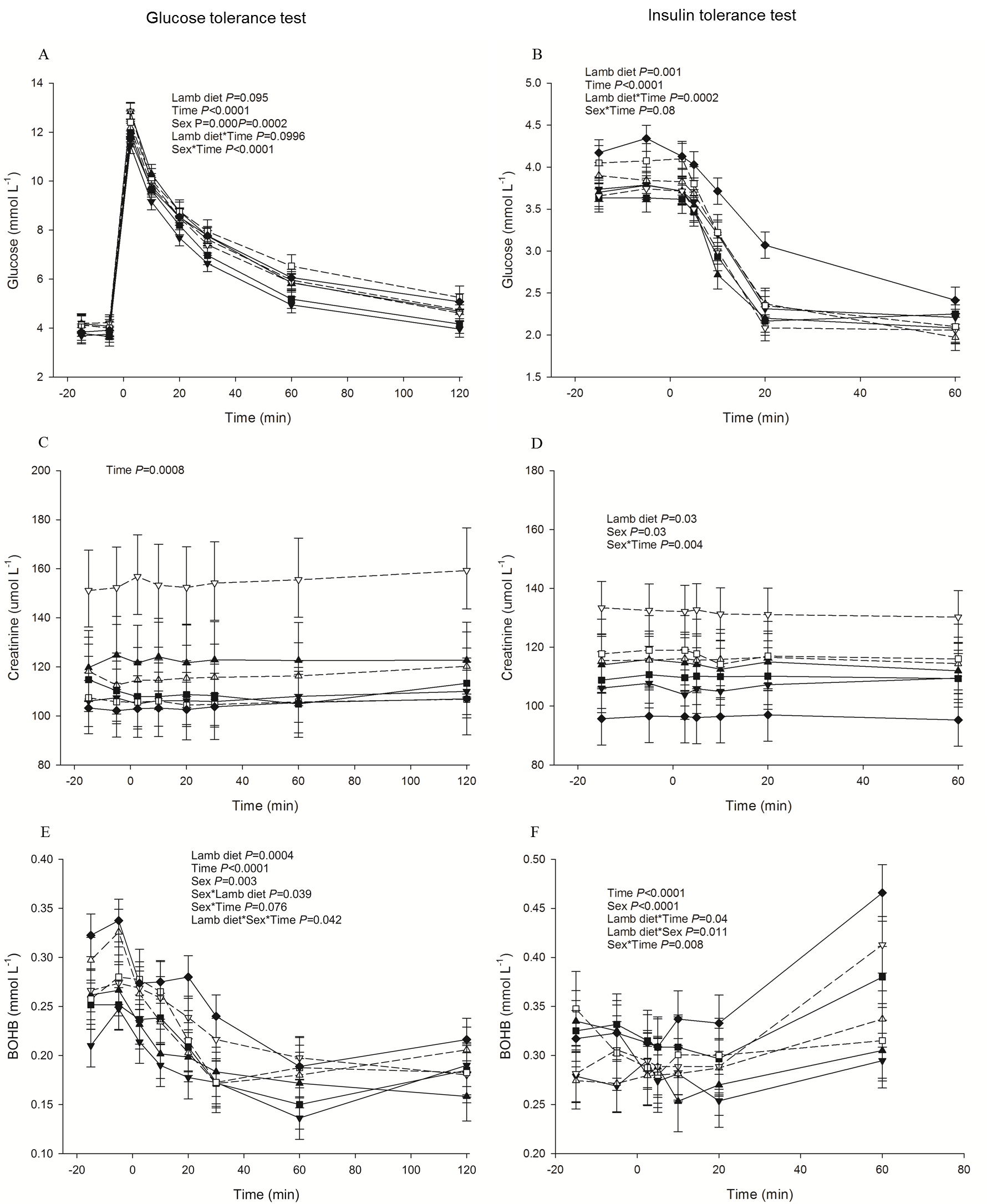

Supplement: S4 Fig — Changes in plasma metabolites during glucose (panels to the left; A, C, E) and insulin (panels to the right; B, D, F) tolerance tests. (A) glucose (B) glucose (C) creatinine (D) creatinine (E) BOHB (β-hydroxy-butyrate) (F) BOHB. Data are shown for each combination of pre- and postnatal diet: LOW-HCHF (N = 7; 4 males, 3 females; dash line, ▽); LOW-CONV (N = 9; 4 males, 5 females; solid line, ▼); HIGH-HCHF (N = 6; 3 males, 3 females; dash line, △); HIGH-CONV (N = 5; 2 males, 3 females; solid line, ▲); NORM-HCHF (N = 4; 2 males, 2 females; dash line, □); NORM-CONV (N = 6; 2 males, 4 females; solid line, ■) and EC (N = 7: 3 males, 4 females; solid line, ♦). HIGH, LOW, NORM, HCHF, CONV and EC: See legends to S1 Fig. (TIF) [file pone.0156700.s004.tif]

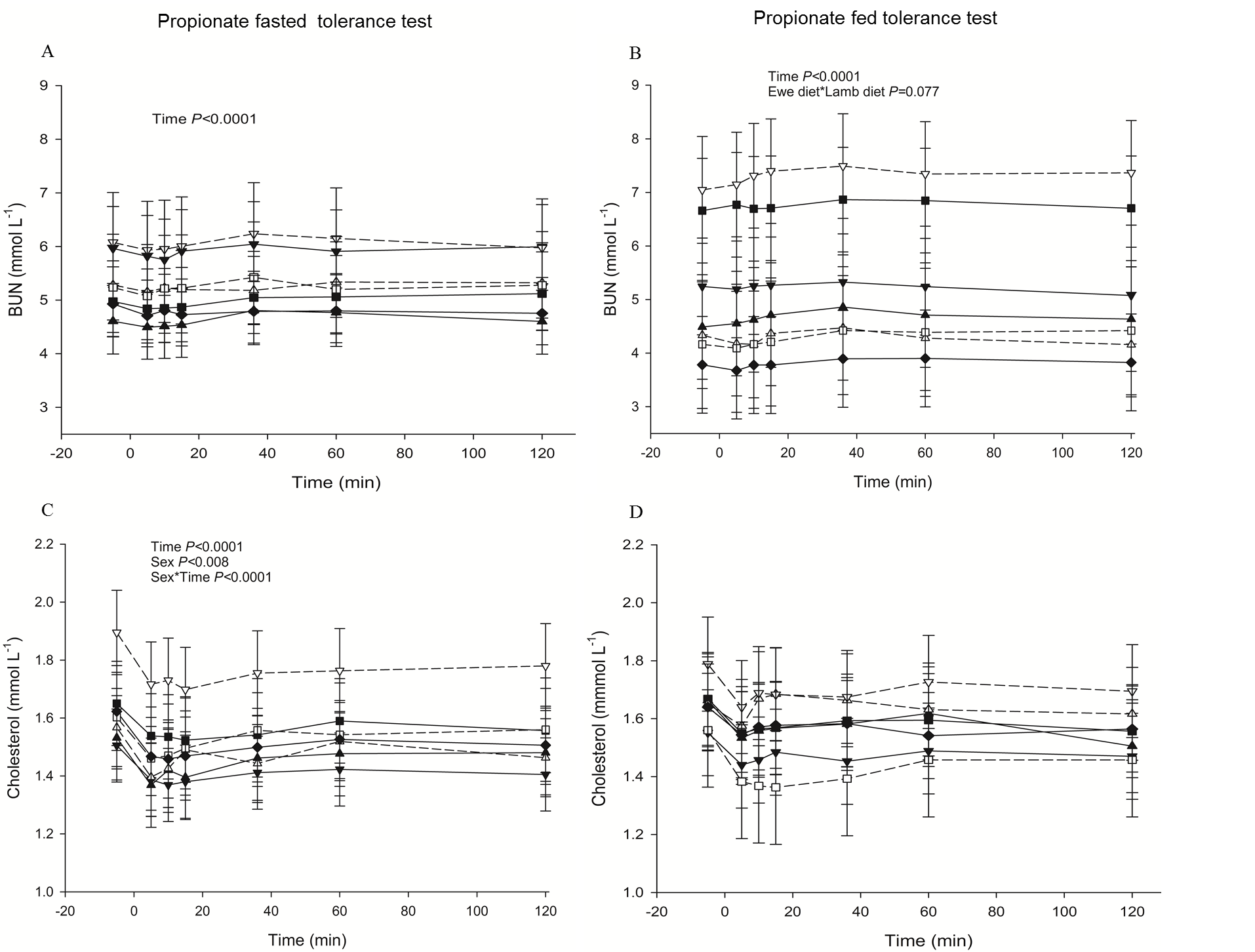

Supplement: S5 Fig — Changes in plasma metabolites during a propionate tolerance test in 68-hour fasted (panels to the left; A, C) and fed (panels to the right; B, D) sheep. (A) BUN (blood urea nitrogen) (B) BUN (C) cholesterol (D) cholesterol. Data are shown for each combination of pre- and postnatal diet: LOW-HCHF (N = 7; 4 males, 3 females; dash line, ▽); LOW-CONV (N = 9; 4 males, 5 females; solid line, ▼); HIGH-HCHF (N = 6; 3 males, 3 females; dash line, △); HIGH-CONV (N = 5; 2 males, 3 females; solid line, ▲); NORM-HCHF (N = 4; 2 males, 2 females; dash line, □); NORM-CONV (N = 6; 2 males, 4 females; solid line, ■) and EC (N = 7: 3 males, 4 females; solid line, ♦). HIGH, LOW, NORM, HCHF, CONV and EC: See legends to S1 Fig. (TIF) [file pone.0156700.s005.tif]
