# Supplementary material for: Long-Term Impacts of Foetal Malnutrition Followed by Early Postnatal Obesity on Fat Distribution Pattern and Metabolic Adaptability in Adult Sheep
Source: PLoS One. 2016 Jun 3;11(6):e0156700. doi: 10.1371/journal.pone.0156700 (PMC4892656; doi:10.1371/journal.pone.0156700)
Supplement: S1 Table — Data are presented as least square means±SEM for organ and tissue weights or expressed as percentage of body weight at 2½ years of age. Fold increase in weights of organs and tissues at 2½ years as compared to 6 months of age has been calculated based on the organ and tissue data from 6 months old lambs which were published previously (Khanal, et al., 2014). NORM (N = 10; 4 males, 6 females), normal diet fulfilling requirements for energy and protein; HIGH (N = 11; 5 males, 6 females), 150% of requirements for energy and 110% of requirements for protein; LOW (N = 16; 8 males, 8 females), 50% of requirements for energy and protein; HCHF (n = 13; 8 males, 5 females), high carbohydrate-high fat diet from birth until six months of age and hay-based normal diet thereafter until 2½ years of age; CONV (N = 13; 7 males, 6 females) conventional diet to achieve moderate and constant growth rates of appr. 225 g day-1 from birth until six months of age and hay-based normal diet thereafter until 2½ years of age; EC, external controls (N = 7; 3 males, 4 females). NORM-CONV (N = 6; 2 males, 4 females); NORM-HCHF (N = 4; 2 males, 2 females); HIGH-CONV (N = 5; 2 males, 3 females); HIGH-HCHF (N = 6; 3 males, 3 females); LOW-CONV (N = 9; 4 males, 5 females); LOW-HCHF (N = 7; 4 males, 3 females); EC (N = 7; 3 males, 4 females). (DOCX) [file pone.0156700.s006.docx]

**S1 Table.** **Changes in fat deposition pattern from adolescence to adulthood after providing moderate diet in all animals**

| **Parameters (actual weight, g or % of total body weight)** | **Age** | **Treatments groups based on the combinations of pre- and postnatal nutrition*** | | | | | |
| --- | --- | --- | --- | --- | --- | --- | --- |
|  |  | **HIGH-HCHF** | **HIGH-CONV** | **LOW-HCHF** | **LOW-CONV** | **NORM-HCHF** | **NORM-CONV** |
| Body weight, kg | 2½-year | 94.7±2.9 | 98.1±3 | 99±2.7 | 91.2±2.4 | 92.1±3.5 | 91.1±2.9 |
|  | Fold increase | 2.5 | 2.7 | 2.4 | 2.6 | 1.9 | 2.4 |
| Subcutaneous fat, g | 2½-year | 540±96 | 686±97.7 | 688.3±88.9 | 554±84.6 | 725.5±117.6 | 535.3±96 |
|  | Fold increase | 2.4 | 15 | 2.1 | 12 | 1.6 | 7.6 |
| Subcutaneous fat % | 2½-year | 0.59±0.09 | 0.71±0.10 | 0.69±0.09 | 0.60±0.08 | 0.78±0.12 | 0.60±0.10 |
|  | Fold increase | 1 | 5.5 | 0.9 | 4.6 | 0.9 | 3.2 |
| Mescenteric fat, g | 2½-year | 3736±524 | 3854±523 | 3698±486 | 2686±453 | 3939±642 | 3019±524 |
|  | Fold increase | 3.2 | 16.8 | 3 | 10.5 | 2.4 | 13.6 |
| Mescenteric fat % | 2½-year | 4.04±0.52 | 4.00±0.57 | 3.69±0.48 | 2.94±0.43 | 4.30±0.64 | 3.37±0.52 |
|  | Fold increase | 1.3 | 6.2 | 1.3 | 4 | 1.4 | 5.9 |
| Perirenal fat, g | 2½-year | 1551±263 | 2542±265 | 1715±245 | 2152±230 | 1888±325 | 1831±265 |
|  | Fold increase | 1 | 15.8 | 0.9 | 12 | 1.1 | 11.8 |
| Perirenal fat % | 2½-year | 1.67±0.27 | 2.65±0.29 | 1.71±0.25 | 2.37±0.22 | 2.07±0.33 | 2.04±0.27 |
|  | Fold increase | 0.38 | 5.8 | 0.37 | 4.7 | 0.6 | 4.9 |

Data are presented as least square means±SEM for organ and tissue weights or expressed as percentage of body weight at 2½ years of age and fold increase in weights of organs and tissues at 2½ years old has been calculated based on the organ and tissue data from 6 months old lambs which are published previously (Khanal, et al., 2014). NORM (N=10; 4 males, 6 females), normal diet fulfilling requirements for energy and protein; HIGH (N=11; 5 males, 6 females), 150% of requirements for energy and 110% of requirements for protein; LOW (N=16; 8 males, 8 females), 50% of requirements for energy and protein; HCHF (n=13; 8 males, 5 females), high carbohydrate-high fat diet from birth until six months of age and hay-based normal diet thereafter until 2½ years of age; CONV (N=13; 7 males, 6 females) conventional diet to achieve moderate and constant growth rates of appr. 225 g day-1 from birth until six months of age and hay-based normal diet thereafter until 2½ years of age; EC, external controls (N=7; 3 males, 4 females). NORM-CONV (N=6; 2 males, 4 females); NORM-HCHF (N=4; 2 males, 2 females); HIGH-CONV (N=5; 2 males, 3 females); HIGH-HCHF (N=6; 3 males, 3 females); LOW-CONV (N=9; 4 males, 5 females); LOW-HCHF (N=7; 4 males, 3 females); EC (N=7; 3 males, 4 females).
