# Supplementary material for: Long-Term Impacts of Foetal Malnutrition Followed by Early Postnatal Obesity on Fat Distribution Pattern and Metabolic Adaptability in Adult Sheep
Source: PLoS One. 2016 Jun 3;11(6):e0156700. doi: 10.1371/journal.pone.0156700 (PMC4892656; doi:10.1371/journal.pone.0156700)
Supplement: S2 Table — Data are presented as least square means±SEM. Values within a row marked by different superscripts are significantly different at P<0.05. For NORM, HIGH and LOW see legends for S1 Table; EC, external controls (N = 7; 3 males, 4 females); M, males; F, females. (DOCX) [file pone.0156700.s007.docx]

**S2 Table.** **Interactive effects of ewe diet and sex on different organ and tissue weights in 2½ year old sheep**

| **Prenatal group** | **HIGH** | | **LOW** | | **NORM** | | **EC** | | ***P* values** |
| --- | --- | --- | --- | --- | --- | --- | --- | --- | --- |
| **Sex** | **M** | **F** | **M** | **F** | **M** | **F** | **M** | **F** | Ewe diet*Sex |
| Body weight, kg | 107.1±2.6^a^ | 88.8±2.2^c^ | 93.3±2.1^bc^ | 96.5±2.2^b^ | 95.6±2.9^bc^ | 88.9±2.4^c^ | 107.6±3.4^a^ | 100.1±2.9^ab^ | 0.0003 |
| Mesenteric fat, g | 3333±521^ab^ | 4125±440^a^ | 1963±412^c^ | 4524±440^a^ | 1987±582^bc^ | 4320±476^a^ | 4108±673^a^ | 3742±582^a^ | 0.03 |
| Mesenteric fat, % | 3.13±0.52^b^ | 4.66±0.44^a^ | 2.11±0.41^b^ | 4.63±0.44^a^ | 2.06±0.58^b^ | 4.86±0.48^a^ | 3.82±0.67^ab^ | 3.71±0.58^ab^ | 0.056 |
| Perirenal fat, g | 1614±237^c^ | 2355±200^b^ | 927±187^d^ | 3116±200^a^ | 707±265^d^ | 2618±216^ab^ | 2245±306^bc^ | 2841±265^ab^ | 0.0009 |
| Peirenal fat, % | 1.50±0.26^cd^ | 2.63±0.22^ab^ | 1.02±0.20^d^ | 3.25±0.22^a^ | 0.74±0.29^d^ | 2.94±0.23^a^ | 2.09±0.33^bc^ | 2.84±0.29^ab^ | 0.008 |
| Pancreas, g | 78.7±10.7^ab^ | 60.6±9.1^b^ | 61.9±9.1^b^ | 79.7±9.1^ab^ | 110.8±12.0^a^ | 67.5±9.8^b^ | 79.2±13.9^ab^ | 62.3±12.0^b^ | 0.0339 |
| Pancreas, % | 0.074±0.01 | 0.069±0.01 | 0.066±0.01 | 0.083±0.01 | 0.118±0.01 | 0.076±0.01 | 0.073±0.01 | 0.063±0.01 | NS |
| Kidney, g | 180±8.9^ab^ | 142±7.5^c^ | 175±7.0^b^ | 173±7.5^b^ | 177±9.9^ab^ | 150±8.1^c^ | 203±11.4^a^ | 155±11.4^bc^ | 0.055 |
| Kidney, % | 0.169±0.011 | 0.161±0.009 | 0.189±0.009 | 0.180±0.009 | 0.186±0.013 | 0.169±0.010 | 0.188±0.014 | 0.154±0.014 | NS |
| Heart, g | 386±19.7^a^ | 295±16.7^b^ | 319±15.6^b^ | 309±16.7^b^ | 387±25.5^a^ | 303±18^b^ | 340±25.5^ab^ | 320±22.1^ab^ | 0.07 |
| Heart, % | 0.36±0.021 | 0.33±0.018 | 0.34±0.017 | 0.32±0.018 | 0.39±0.028 | 0.34±0.019 | 0.32±0.027 | 0.32±0.023 | NS |

Data are presented as least square means±SEM. Values within a row marked by different superscripts are significant different at *P*<0.05. For NORM, HIGH and LOW see legends for Supplementary table 1; EC, external controls (N=7; 3 males, 4 females); M, males; F, females.
